# Chirality-Induced Spin Selectivity: A Minimal Model

**DOI:** 10.1021/acs.jpclett.5c01813

**Published:** 2025-08-26

**Authors:** Lorenzo Savi, Leonardo Celada, D.K. Andrea Phan Huu, Alessandro Chiesa, Stefano Carretta, Anna Painelli

**Affiliations:** † Department of Chemistry, Life Science and Environmental Sustainability, 9370 University of Parma, Parma 43124, Italy; ‡ Department of Mathematical, Physical and Computer Sciences University of Parma,Parma 43124, Italy; ⊥ INFN-Sezione di Milano Bicocca, Gruppo Collegato di Parma, 43124 Parma, Italy

## Abstract

Chirality-induced spin selectivity (CISS) is an intriguing yet poorly understood phenomenon observed when electrons travel through a chiral medium or molecule. We propose a current-constrained approach to drive a current through a linear Hubbard chain of twisted *p* orbitals, thus simulating the electron motion through a chiral molecular system. In this original approach, CISS can be addressed in systems with correlated electrons coupled to nonadiabatic molecular vibrations. Sizable CISS responses are obtained in some parameter space, with a clear amplification of CISS in non-half-filled systems. Peierls vibrations play a special role: CISS cannot be observed in a Hubbard chain in the lack of next nearest neighbor interactions, but an out-of-equilibrium stretching mode can lead to finite polarization even in these systems.

## Graphical abstract

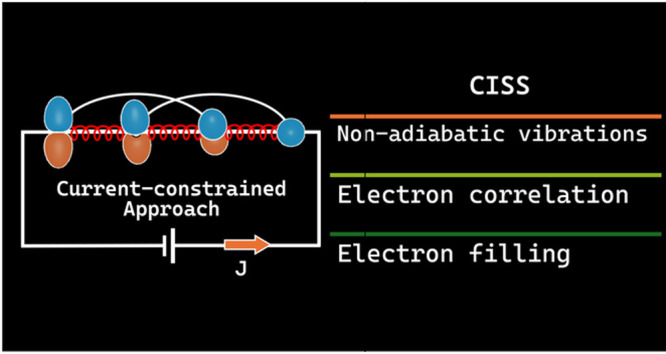

Chirality, the property of systems not superimposable to their specular image, is intrinsically connected to symmetry:^1^ chiral systems do not possess a roto-reflection axis of any order, including reflection planes and inversion centers. The optical and spectroscopic consequences of chirality are well-known, and circular dichroism, the differential absorption of left and right polarized light, is a well established technique to address chirality.^2^ Enantioselective interactions, the preferential interaction between enantiomeric species, are well-known in chemistry, providing the basis for asymmetric synthesis.^3–8^ The electron spin itself is nonchiral, but a moving electron carrying a spin is chiral^1^ and therefore can specifically interact with chiral molecules. This is the hearth of the phenomenon dubbed *chirality induced spin selectivity* (CISS), that describes the differential transmission of spin up and spin down electrons traveling through a chiral medium.^9^ CISS was observed when photoemitted electrons travel through layers of chiral molecules,^10^ when a current is driven through chiral self-assembled monolayers^11^ or through single chiral molecules,^12^ and, more recently, when an electron travels through a molecule via photoinduced electron-transfer.^13–15^ The issue here is not the observation of CISS, that is predictable in terms of symmetry considerations, the astonishing and so far not fully understood observation is the amount of CISS, leading to spin polarization well beyond 50%.

CISS requires the breaking of time-reversal symmetry, as guaranteed by traveling electrons, as well as the breaking of the spin-degeneracy, as made possible by spin orbit coupling (SOC).^16^ In organic molecules, SOC is tiny,^9,17^ and several strategies for its amplification, as needed to explain the large spin polarizations observed experimentally, have been proposed, relying on electron–electron interactions,^16,18,19^ electron-vibration coupling,^20,21^ quantum interference,^22^ polaronic effects,^23–25^ geometrical curvature in helical atomic chains,^26,27^ the close proximity to conical intersections,^28–30^ and dephasing phenomena,^16,31^ to cite just a few examples.

Modeling CISS is nontrivial not only because of the tiny and somewhat elusive nature of SOC but also because it requires addressing traveling electrons. In order to observe CISS, approaches must be devised to drive electrons through the system either running an electric current or via photoexcitation. Of course, the outcome of the simulation will vary depending on the way the system is prepared, giving rise to a large number of different approaches with sometimes contrasting results. Here, we propose forcing the electron motion through the system exploiting a strategy that, to the best of our knowledge, has not been applied to CISS as yet. Specifically, we rely on a current-constrained (CC) approach, which imposes a current through a system via the mathematical trick of Lagrange multipliers,^32–34^ offering an easy and flexible way to mimic the electron motion through a molecule. Unlike conventional nonequilibrium Green’s function methods (NEGF)^35,36^ that impose open boundary conditions through reservoir self-energies and often employ Büttiker probes to simulate dephasing and spin-flip scattering on an energy and bias-resolved basis,^37^ the CC formalism retains a closed-circuit, many-body perspective by enforcing a prescribed steady-state current through Lagrange multiplier constraints on bond-current operators. This choice obviates the need to specify chemical potentials or Fermi distributions for explicit leads and probes, embedding the nonequilibrium within the variational ground state of the augmented Hamiltonian. As a result, the CC formalism requires only the explicit form of the system Hamiltonian to compute the steady-state current, obviating the need to derive reservoir self-energies for each interaction, allowing one to explore regimes of strong interaction, such as photodriven currents or persistent loop currents within a unified computational framework. Although NEGF approaches offer direct control over bias voltage, temperature, and dephasing strength through adjustable self-energies and probe couplings,^38^ the CC method trades this flexibility for a direct handle on the current magnitude, with the effective voltage drop inferred via phenomenological relaxation parameters. CC provides complementary insights into correlation-driven spin polarization phenomena without recourse to explicit reservoirs and is therefore particularly well suited for the theoretical investigation of correlated transport in chiral systems.

As for the system, we consider the simplest model for correlated electrons, a linear Hubbard chain composed of *N* (typically 4) sites along the *z* axis. Structurally, the system is nonchiral, but electronic chirality, or electrohelicity,^39,40^ is introduced accounting on each site for a single *p*-type orbital, perpendicular to *z*, but twisted along the chain so that the orbital on site *i* + 1 is rotated in the *xy* plane by an angle θ_*i*_ vs the orbital on site *i*, as shown in Figure 1a. Our results show that the large spin polarization observed experimentally can hardly be recovered in simple models for correlated electrons. They hint, however, to amplified CISS responses in nonhalf-filled systems and/or in the presence of weak bonds. Moreover, they point to a key role played by vibronic coupling in CISS, with the strong constraint that coupled vibrations are kept out of equilibrium.

**Figure 1:**
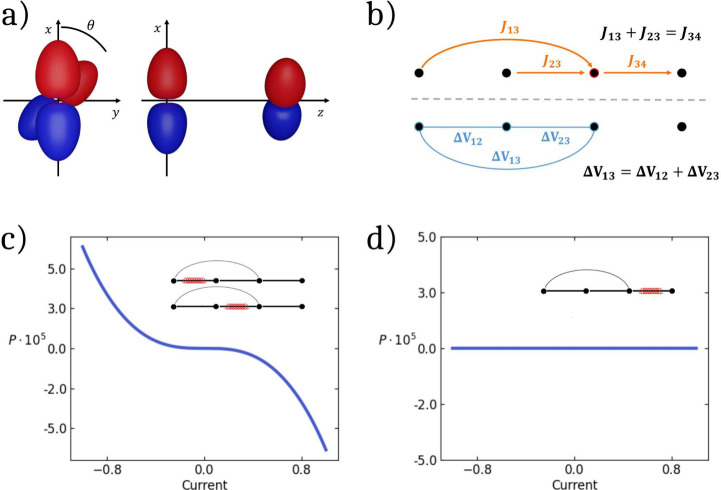
(a) Schematic front and side views of two adjacent *p* orbitals. (b) Red: illustration of the constraint on the currents through site 3 to avoid charge accumulation; blue: illustration of the constraint on the potential drop. (c and d) The current-dependent polarization for a four site chain with *U* = 5, ϵ_1_ = – ϵ_4_ = 2.5, *t*_12_ = *t*_23_ = 1, *t*_13_ = 0.01, and χ = 0.01. The insets show the relevant connectivity with black lines marking the electron-hopping channels, and the red helix marking the bond where SOC is introduced. The two structures depicted in panel (c) lead to the same result. The structure in panel (d) leads to vanishing polarization.

The extended Hubbard Hamiltonian reads:

$$H_{Hu}=\overset{N}{\underset{i=1}{\sum }}\epsilon_{i}{\mathrm{n̂}}_{i}-\underset{i<j}{\sum }t_{ij}{\mathrm{b̂}}_{ij}+\overset{N}{\underset{i=1}{\sum }}U_{i}{\mathrm{n̂}}_{i\alpha }{\mathrm{n̂}}_{i\beta }+\underset{i<j}{\sum }W_{ij}\left(Z_{1}-{\mathrm{n̂}}_{i}\right)\left(Z_{i}-{\mathrm{n̂}}_{j}\right)$$ 1

In the above equation, *n̂*_*i*_ = *n̂*_*iα*_ + *n̂*_*i*,β_ counts the total number of electrons on site *i*, *n̂*_*iσ*_ = *a*_*iσ*_^†^ *a*_*iσ*_ counts the electrons with spin σ on the same site and *a*_*iσ*_^†^ (*a*_*iσ*_) creates (annihilates) an electron with spin σ = α, β on site *i*. The operator *b̂*_*ij*_ = *b̂*_*ijα*_ + *b̂*_*ijβ*_, with *b̂*_*ij*,σ_ = (*a*_*iσ*_^†^ *a*_*jσ*_ + *H*.*c*.), measures the bond-order between sites *i* and *j*. The on-site energy is ϵ_*i*_, *U*_*i*_ is the repulsion between two electrons on the same site, *W*_*ij*_ measures the interaction between charges on sites *i* and *j*, and *Z*_*i*_ is the charge on the *i*-th core, i.e. the charge residing on the site when all electrons explicitly accounted for in the Hubbard Hamiltonian are removed. In the following, we will show results obtained for *W*_*ij*_ = 0, while selected results for finite intersite electronic interactions are shown in the ESI.

We introduce SOC interactions accounting for the one-electron term of the Breit-Pauli Hamiltonian:^41^

$$H_{SOC}=\frac{1}{2m_{e}^{2}c^{2}}\underset{r}{\sum }\underset{i}{\sum }\frac{1}{r_{ri}^{3}}l_{ri}\cdot s_{r}$$ 2

where ***s***_*r*_ is the spin angular momentum of electron *r*, **l**_*ri*_ is its angular momentum with respect to the nucleus on site *i* and *r*_*ri*_ is the distance of the *r* electron from the *i* nucleus. The cubic distance in the denominator suggests that the dominant terms describe the interaction of an electron residing at site *i* with the *i*-th nucleus, so longer distance contributions are neglected. With this approximation (see ESI Section S1) the SOC Hamiltonian becomes

$$H_{SOC}=i\underset{i<j}{\sum }\chi_{ij}\left({\mathrm{v̂}}_{ij,\alpha }-{\mathrm{v̂}}_{ij,\beta }\right)$$ 3

where χ_*ij*_ measures the strength of the SOC interaction between electrons residing on sites *i* and *j* and

$${\mathrm{v̂}}_{ij,\sigma }={â}_{i,\sigma }^{†}{â}_{j,\sigma }-\mathrm{H.c.}$$ 4

is the velocity dipole operator. In the adopted model, the electron orbital momentum is aligned along the chain and the SOC interaction necessarily preserves the *z* component of the total spin, *Ŝ*_*z*_, so that spin-flips are not accounted for. This is an inherent feature of the proposed model, but investigating the role of spin flips in CISS would definitely be interesting, particularly in view of the contrasting opinions in recent literature.^16,42^ A real-space basis will be adopted, with each basis state corresponding to a configuration obtained distributing the electrons on the sites. Since in the adopted model [*Ŝ*_*z*_, *Ĥ*] = 0, state with different ⟨*Ŝ*_*z*_⟩ stay unmixed and, limiting attention to systems with an even number of electrons, we work in the *S*_*z*_ = 0 subspace (more details in Section S2).

To describe electrons moving across the Hubbard chain, we rely on the CC approach.^32–34,43–46^ Specifically, to enforce a current *J* through the system, a set of Lagrange multipliers, λ_*lm*_, enters the Hamiltonian, as follows:

$$H\left(J\right)=H-\overset{1,N-1}{\underset{l}{\sum }}\overset{l+1,N}{\underset{m}{\sum }}\lambda_{lm}{ĵ}_{l}^{m}$$ 5

where *ĵ*_*l*_^*m*^ is the operator that measures the current flowing from site *l* to site *m*. The Lagrange multipliers are optimized so that in the ground state of the current carrying system, |*G*(λ)⟩, the incoming flux through the first site and the outcoming flux through the last site are equal to the current imposed on the system, *J*:

$$J=\overset{N}{\underset{i=2}{\sum }}\left\langle G\left(\lambda \right)\right|{ĵ}_{1}^{i}\left|G\left(\lambda \right)\right\rangle =\overset{N-1}{\underset{i=1}{\sum }}\left\langle G\left(\lambda \right)\right|{ĵ}_{i}^{N}\left|G\left(\lambda \right)\right\rangle$$ 6

while ensuring a balanced incoming and outcoming flux on all other sites, and hence enforcing a steady-state current without charge accumulation:

$$\overset{k-1}{\underset{l=1}{\sum }}\left\langle G\left(\lambda \right)\right|{ĵ}_{l}^{k}\left|G\left(\lambda \right)\right\rangle -\overset{N}{\underset{l=k+1}{\sum }}\left\langle G\left(\lambda \right)\right|{ĵ}_{k}^{l}\left|G\left(\lambda \right)\right\rangle =0$$ 7

The constraints in eqs 6 and 7 fully define the Lagrange multipliers in a system with only nn hopping and SOC, but, in the presence of long-range hopping and/or SOC, another constraint is needed to ensure that the potential drop between two nodes is the same for different paths (cf Figure 1b). This brings us to the delicate issue of the relationship between the Lagrange multipliers and the potential drop. As discussed in ref.,^34^ the relation can be drawn by calculating the power spent on the system to maintain the current. Since the current is an off-diagonal operator on the eigenstates of the unperturbed Hamiltonian, the power spent on the system is governed by the relaxation of the off-diagonal elements of the density matrix, the coherences. In the simplifying hypothesis of a system with large inhomogeneous broadening, where the same relaxation time, 1/Γ, can be applied to all coherences, the potential drop in each bond is simply proportional to the relevant Lagrange multiplier, *V*_*ij*_ = Γλ_*ij*_. Although this approximation can be relaxed, we will adopt it to keep the model as simple as possible. Accordingly, the Lagrange multipliers on nnn bonds are set as the sum of the two relevant nn Lagrange multipliers: λ_*i*,*i*+2_ = λ_*i*,*i*+1_ + λ_*i*+1,*i*+2_.

The standard definition of the current operator, in terms of the velocity dipole,^47,48^ *ĵ*_*i*_^*j*^ ∝*v̂*_*ij*_ = *v̂*_*ijα*_ + *v̂*_*ij*,β_, leads to nonphysical results, as illustrated in ESI (Figure S1). A correct definition of the current operator must properly account for SOC. Specifically, the flux of electrons through site *k*, calculated as the time derivative of *n̂*_*k*_, is the sum of incoming currents from all sites connected to *k* from the left side minus the sum of all currents exiting from site *k* toward all connected sites to the right of *k*, as sketched in Figure 1b. Accordingly:

n˙^k=i[H,n̂k]=∑i1,k−1ĵik−∑ik+1,Nĵki 8

where we use units with *ℏ* = 1 and

$${ĵ}_{l}^{m}=it_{lm}\left({\mathrm{v̂}}_{lm\alpha }+{\mathrm{v̂}}_{lm\beta }\right)-\chi_{lm}\left({\mathrm{b̂}}_{lm\alpha }-{\mathrm{b̂}}_{lm\beta }\right)$$ 9

Then SOC explicitly enters the definition of the current operator, with a contribution that has opposite sign for α and β electrons.

Having a model to drive electrons through a chiral Hubbard chain, we can address spin-selective behavior. We impose that charges do not accumulate in the current-carrying system. However, if the mobility of electrons depends on the spin, spin density can accumulate on the sites. The on-site spin-density is defined as ρ_*i*_ = ⟨*n̂*_*iα*_ – *n̂*_*iβ*_⟩ and the spin polarization across the system reads

$$P=\frac{\rho_{N}-\rho_{1}}{2}$$ 10

A second approach to address CISS mimics transport experiments, where a magnetized electrode is used to inject spin polarized electrons in a junction. To simulate this experiment, we use the same trick of the Lagrange multiplier to impose a current of electrons with α spin:

$$H\left(J_{\alpha }\right)=H-\underset{i}{\sum }\lambda_{i,\alpha }{ĵ}_{i,\alpha }$$ 11

Then we do the same for β electrons. Of course, due to CISS, different potentials are needed to sustain the α or β-polarized current, or, in other terms, different spin-currents are calculated for the same potential drop. Accordingly, for each value of the total potential drop, we estimate the CISS efficiency as *G* = (*J*_α_ – *J*_β_)/(*J*_α_ + *J*_β_). In the literature, this quantity is typically referred to as spin-polarization. Here, in order to avoid confusion with the spin polarization defined in eq 10, we dub it as *current anisotropy*. It is important to stress that addressing the current anisotropy along these lines may only work in models where SOC does not flip the spins. In these conditions, the steady-state flux of spin-polarized electrons imposes a continuity relation on the spin current, a condition that would be broken in the presence of spin-flip events.

It is well-known that spin polarization cannot be supported in a linear chain featuring only nn interactions.^49^ In Section S4 of ESI we demonstrate that this result holds true also for current-carrying systems. Often, to allow for finite polarization, next-nearest-neighbor (nnn) SOC interactions are introduced,^18,19^ following an original suggestion by Kane and Mele for graphene-based systems.^50,51^ However, spin polarization can be observed in a linear chain without introducing nnn SOC, but accounting for nnn hopping, as illustrated in Figure 1c,d (see also ESI, section S5). Quite interestingly, a single nn SOC interaction in the current-carrying chain is enough to enable spin polarization, provided that it operates inside a loop generated by the electron hopping interactions, in a situation strongly reminiscent of the persistent spin currents induced by SOC in mesoscopic rings.^52^ In the following, we will discuss results obtained introducing a tiny nnn hopping (typically 2 orders of magnitude smaller than the nn hopping); results obtained introducing nnn SOC are marginally different. To support a net spin polarization, nonequivalent sites must be present in the chain. Therefore, we will present results relevant to systems where the energy of the last and first sites differ by 2Δ = ϵ_*N*_ – ϵ_1_, while setting all other on-site energies to zero. Of course, a system with Δ = 0 supports finite on-site spin densities and may sustain finite current anisotropy, as shown in Figure S3.

Before proceeding, it is important to illustrate how the relevant dimensionless quantities, current and potential drop, in the figures translate into dimensional quantities. The dimensionless current should be multiplied by *et*/*ℏ* to get dimensional values. Then, setting e.g. *t* ∼ 0.1 eV, a dimensionless current *J* = 1 would correspond to ∼ 20 μA. The voltage drop should in turn be multiplied by *ℏ*Γ/*e*. Setting 1/Γ ∼ 100 fs as a typical lifetime of electronic states, λ = 1 would correspond to a potential drop ∼ 6 mV. For comparison, in a recent single molecule experiment,^12^ current of the order of ∼ 1 μA are obtained for potential drops of the order of 1 V.

Figure 2a shows results for a half-filled Hubbard chain with the nn hopping set to 1 as the energy unit, nnn hopping and nn SOC set to 0.01, *U* = 5 and variable Δ. In this system, a finite polarization is observed, which, however, remains very small, the largest value being reached for *U* ∼ 2Δ, where the current anisotropy also reaches its maximum value. Results for other *U* values and also including intersite electron–electron interactions are shown in ESI, Figures S4–S7. The calculated polarization amplitudes and current anisotropies are orders of magnitude smaller than reported by experimentalists. Increasing the nnn hopping helps to increase the polarization and the current asymmetry (see Figure S8), but again estimated values are far too small if compared with experiment.

**Figure 2:**
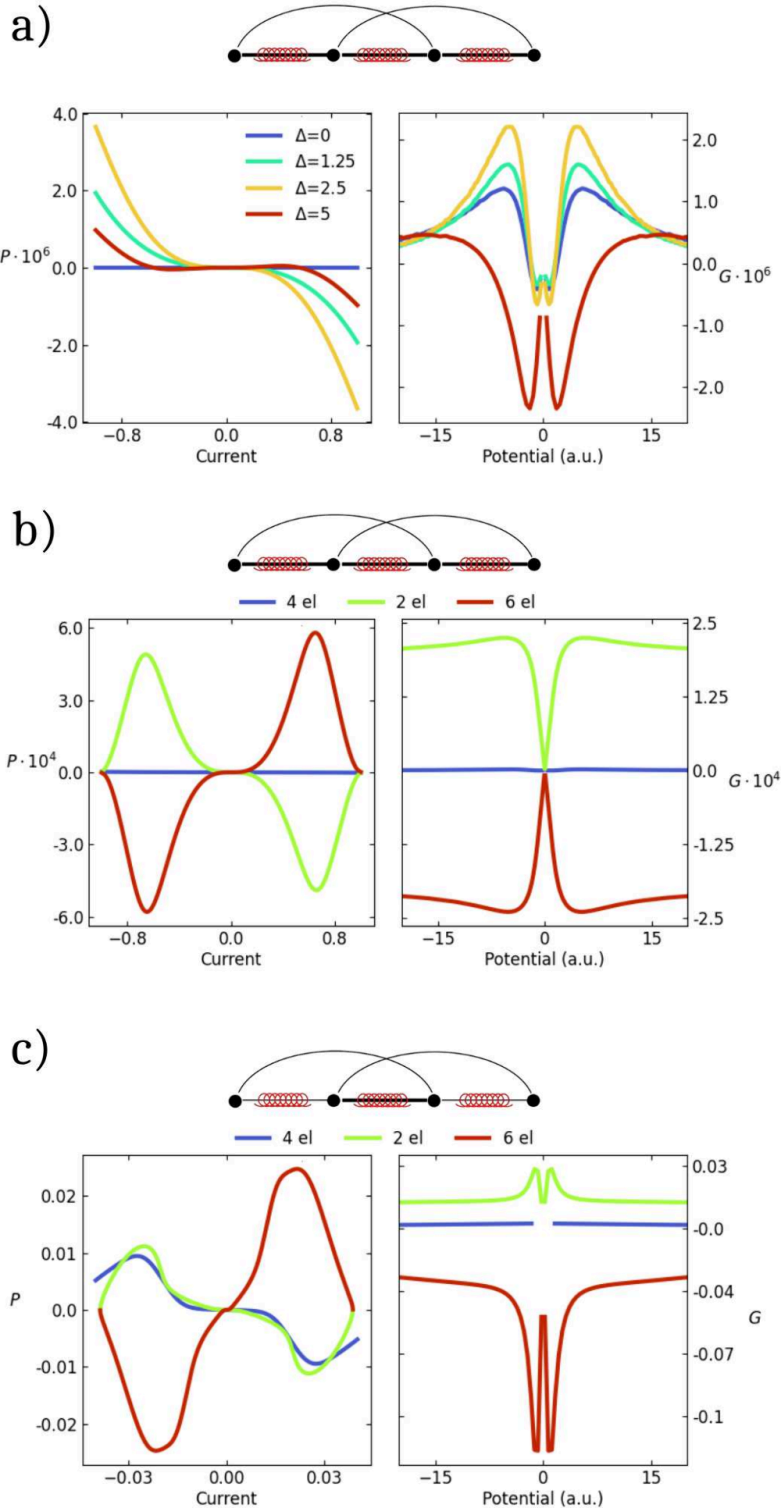
All panels: the 4 sites Hubbard chain with *U* = 5. Left: polarization vs current; Right: current anisotropy vs the voltage. (a) Results are shown for nn hoppings *t* = 1 and SOC χ = 0.01, nnn hoppings *t′* = 0.01, and variable Δ. (b) Results for 2Δ = 5, nn *t* = 1 and χ = 0.01, nnn *t′* = 0.01. The blue curve refers to the half-filled case, the green and the red curves refer to a system with 2 and 6 electrons, respectively. (c) Results for 2Δ = 5, nn hoppings: *t*_23_ = 1, *t*_12_ = *t*_34_ = 0.01, nnn *t′* = 0.01 and nn χ = 0.01. The blue curve refers to the half-filled case, the green and the red curves refer to a system with 2 and 6 electrons, respectively.

Moving away from half-filling is an interesting possibility toward amplified responses. Figure 2b shows a clear amplification of the responses for the same system as in Figure 1a for 2Δ = *U* = 5 when the number of electrons is either decreased from 4 to 2 or increased to 6 (results for different values of model parameters are shown in Figures S9 and S10). The electron–hole symmetry is clearly broken in a system with finite Δ, and the 1/4 and 3/4 filled systems show slightly different behavior (see also Figure S11 for additional details). Introducing weak bonds also helps. Specifically, if we consider largely distorted bond with θ ∼ π/2, the relevant hopping integral decreases, reaching values of the same order of magnitude as χ. In Figure 2b the blue line shows results for a chain with *U* = 2Δ = 5, and where the lateral bonds are highly distorted as to set the relevant χ = *t* = 0.01. Here the polarization reaches values up to 1%. Bringing together the two amplification strategies finally leads to the results in Figure 2c, where the spin polarization and current anisotropy reach values up to a few percent and to 10%, respectively. The observed amplification is much larger in this case for the 3/4 filled system than for the 1/2 filled chain. Less satisfying, yet qualitatively similar results are shown in Figure S12, for a system where the central bond is weak.

Reaching a few percent of polarization or of current anisotropy is certainly encouraging. However, this result does not solve the CISS conundrum. Finding sizable polarization in a special parameter range does not fully explain CISS, a phenomenon that is observed in a very wide range of materials governed by qualitatively different physics. More robust results are needed, which apparently are hardly recovered in simple models for correlated electrons, a result that, in a different context, has been recently underlined.^53^ On the positive side, results in Figure 2c could explain the large current anisotropies measured in transport measurements: the number of electrons inside the molecular junction depends on the circuit details,^54^ and it is likely that in the steady-state regime the junction bears a number of electrons different from the isolated molecule. We notice that the spin polarization has a smooth evolution with the current, while the current anisotropy shows anomalies around *V* = 0. This anomaly is intrinsic to a quantity whose denominator vanishes at *V* = 0. Strong nonlinearities of *G* around *V* = 0 have indeed been experimentally observed.^55–58^

Vibrations are often discussed as a possible source of spin polarization.^24,59–61^ In the CC approach, we can address nonadiabatic vibrations in a correlated electron model. A unitary transformation allows to embed the Holstein vibrations, modulating on-site energies, into the Hubbard Hamiltonian via a renormalization of model parameters (see ESI, section S8). Accordingly, spin polarization cannot be switched on by Holstein coupling when electrons travel in systems with only nn hopping and SOC terms. More generally, introducing Holstein modes in models with nnn hopping has marginal effects on the polarization, basically amounting to a renormalization of the on-site energies, ϵ_*i*_ and *U*_*i*_ (see ESI, Section S8).

The unitary transformation does not work for Peierls vibrations, leading to more interesting results. A Peierls mode modulating the coupling between sites *l* and *m* adds a vibronic term to the electronic Hamiltonian as follows:

$${Ĥ}_{vib}=\hbar \omega_{\nu }\left({â}_{\nu }^{†}{â}_{\nu }+\frac{1}{2}\right)+g_{\nu }\left({â}_{\nu }^{†}+{â}_{\nu }\right){\mathrm{b̂}}_{lm}$$ 12

where *â*_ν_^†^, *â*_ν_ are boson creation and annihilation operators, respectively, relevant to a mode with frequency ω_ν_. The vibrational mode modulates the *l* – *m* hopping with a coupling constant *g*_ν_, the corresponding relaxation energy being ϵ_ν_ = *g*_ν_^2^/ω_ν_. The same vibration could also modulate the SOC, but the effect of SOC modulation is marginal. It must be recognized that if Peierls coupling is introduced, the current operator must be modified accordingly. In line with eq 9 the current operator becomes

$${ĵ}_{l}^{m}=i\left[t_{lm}-g_{\nu }\left({â}_{\nu }^{†}+{â}_{\nu }\right)\right]\left({\mathrm{v̂}}_{lm\alpha }+{\mathrm{v̂}}_{lm\beta }\right)-\chi_{lm}\left({\mathrm{b̂}}_{lm\alpha }-{\mathrm{b̂}}_{lm\beta }\right)$$ 13

The vibronic Hamiltonian (including electron–electron interactions) is written on the basis obtained as the direct product of the real-space electronic basis (see section S2) times the eigenstates of the harmonic oscillator(s), truncating the vibrational basis to a large enough number of vibrational quanta to obtain converged results. Direct numerical diagonalization leads to numerically exact nonadiabatic results for the current-carrying system. If several vibrational modes are introduced, the base grows rapidly, making the calculation computationally intensive. In the following, we will only address systems where a single mode is considered. Quite interestingly, we present for the first time results relevant to a system where electron–electron and nonadiabatic Peierls vibrations are accounted for at the same time.

Figure 3a shows results for a four site Hubbard chain with nn *t* = 1 and χ = 0.01, nnn *t′* = 0.01, and *U* = 2Δ = 5. A Peierls mode with frequency ω_ν_ = 0.1 modulates the central hopping integral, with relaxation energy increasing from 0 to 0.1. The results are disappointing: both the polarization and the current anisotropy monotonously decrease upon increasing the strength of Peierls coupling (see Figure S13 for analogous results for *U* = 0). Similar results are obtained accounting for a vibrational mode that modulates either symmetrically or antisymmetrically the two lateral bonds (see ESI, Figures S14, S15, S16 and S17).

**Figure 3:**
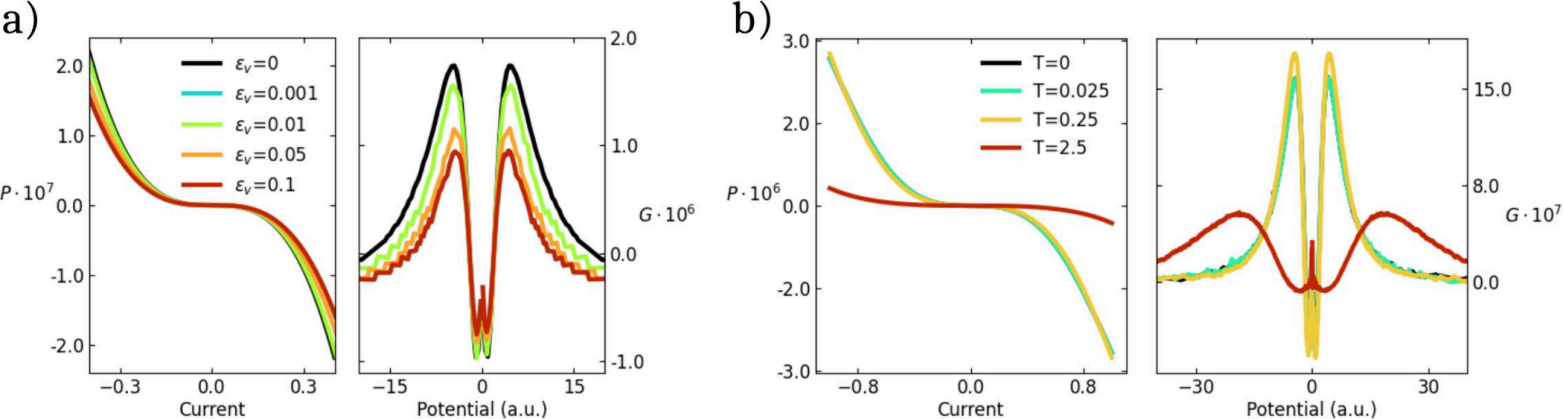
Half-filled 4-site Hubbard chain with nn hopping *t* = 1, nnn hopping *t′* = 0.01, χ = 0.01, *U* = 2Δ = 5. A Peierls mode modulates the hopping integral between the two central sites with ω_ν_ = 0.1. (a) The spin polarization vs the current and the current anisotropy vs the potential drop calculated for different values of ϵ_*ν*_. (b) The same quantities as in (a) are shown for different temperature for the same system in (a) with ϵ_ν__*v*_ = 0.1.

The calculation can be extended to finite temperature, calculating all expectation values accounting for the Boltzmann population of the states. Figure 3b shows results for the same system in Figure 3a and ϵ_*ν*_ = 0.1. The temperature has marginal effects as long as it stays lower than the singlet–triplet gap (0.7 in the specific case), but both the polarization and the current anisotropy are suppressed at higher temperatures (see also Figure S18).

Peierls vibrations apparently do not lead to major effects, and noteworthy they do not relax the strict requirement of nnn interactions to observe CISS. Indeed, we checked explicitly that driving a current in systems with either Holstein or Peierls coupling always leads to vanishing polarization, as long as nnn interactions are disregarded. This result contrasts sharply with several reports showing CISS, and often quite substantial spin polarization effects, in two-site systems (where nnn are obviously missing) or in systems with only nn hopping and SOC but in the presence of Peierls coupling.^20,53,61–64^

In the CC approach, the steady-state current is driven through a system where vibrations are always maintained in equilibrium. This represents a qualitative difference vs other approaches. To demonstrate this point, we consider the simplest system, a two-site Hubbard model, with Peierls coupling. As stated above, if the current operator is properly defined and the CC approach is adopted with equilibrated vibrations, CISS responses exactly vanish. However, a different picture emerges if, in the same spirit as in ref^20^ we adopt a mean-field approach to vibrations, basically blocking the coordinate to its equilibrium value in the system without current *Q*_0_ = ⟨*â_ν_* ^†^ + *â_ν_* ⟩_0_. This approach could be applied to vibrational modes that are slow enough not to be able to readjust during the current flow.

Technically, to fix the vibrational coordinate to *Q*_0_ we introduce an additional Lagrange multiplier in the Hamiltonian, as follows:

$$H\left(J,\alpha \right)=H_{vib}-\overset{1,N-1}{\underset{l}{\sum }}\overset{l+1,N}{\underset{m}{\sum }}\lambda_{lm}{ĵ}_{l}^{m}-\alpha \left(\left\langle {â}_{v}^{†}+{â}_{v}\right\rangle -Q_{0}\right)$$ 14

Figure 4a collects results for a two-site Hubbard chain with *t* = 1 as the energy unit, χ = 0.01, *U* = 2Δ = 5, ϵ_*ν*_ = 0.1 and increasing frequency (or coupling constant). The right panel shows how the equilibrium value of the vibrational coordinate varies with the current in the fully equilibrated approach. In these conditions, the calculated spin polarization exactly vanishes for any value of the current (black line in the left panel of Figure 4a). In the left panel of Figure 4a, colored lines show instead the polarization calculated as a function of the current imposing that, regardless of the current, the expectation value of the vibrational coordinate is fixed to *Q*_0_. In this case, the spin polarization is finite and increases with the current and with the strength of the coupling.

**Figure 4:**
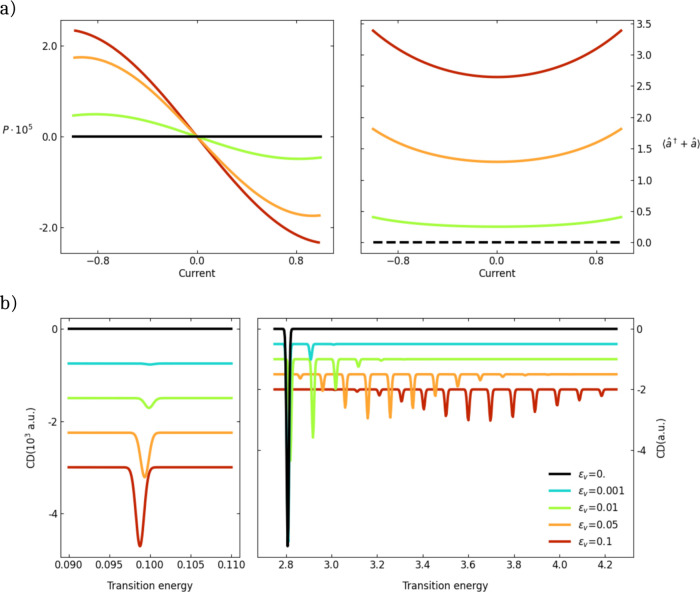
(a) The half-filled 2-sites Hubbard model with *t* = 1, χ = 0.01 and *U* = 2Δ = 5. A Peierls mode with frequency ω_ν_ = 0.1 modulates the hopping integral between the two sites with different *ε*_*ν*_ as per the legend in panel (b). The left panel shows the polarization against the current calculated with the phonon coordinate constrained to its equilibrium value in the absence of current. The right panel shows the equilibrium value of the coordinate vs the current. (b) Circular dichroism spectra (arbitrary units) for the same systems as in (a) without current. The two panels refer to the spectral region of vibrational (left) and electronic (right) transitions. The intensity of the spectra in the left panel are multiplied by a factor 10^3^ for clarity.

Although the spin polarization remains small, the qualitative effect of out-of-equilibrium vibrational modes in allowing for finite spin-polarization even in the absence of nnn interactions is interesting, as it points to a major role of vibrations in CISS. Results in Figure 4 refer to a two-site Hubbard chain, a structurally nonchiral system, where chirality is enforced by the orbital twist. In this system, the stretching mode modulates the hopping integral and hence is not directly coupled to the spin degrees of freedom. Quite interestingly, the stretching mode, which by itself is clearly nonchiral, acquires a chiral nature due to the coupling to the electronic system, as demonstrated in Figure 4b that shows circular dichroism spectra calculated for the same systems as in Figure 4a in the absence of current (see Section S10 for computational details).

Circular dichroism, measuring the differential absorption of left and right circularly polarized light,^2^ is a clear signature of chirality. In the region of the electronic transitions (right panel of Figure 4b) Peierls coupling shows up, as expected, with the appearance of vibronic bands: besides the 0–0 band, the 0–1, 0–2 etc become progressively more intense upon increasing the strength of the coupling. More interesting is the region of vibrational transitions, where a signature appears in the circular dichroism spectrum whose intensity, while staying a few orders of magnitude smaller than for the electronic transition, increases with the coupling strength, while the relevant transition energy slightly decreases. In chiral molecules, the chiral responses of vibrational states are well-known and widely investigated;^65,66^ our results suggest that vibrational modes in chiral molecules may play a major role in CISS.

We have presented an original discussion of CISS-related phenomena, where a CC approach is adopted to force the electron motion through a chiral Hubbard chain. The approach is simple and allows us to explore large regions of parameter space. As expected, as long as only electronic degrees of freedom are considered, CISS is supported by electrons traveling along a simple Hubbard chain only if nnn hopping or SOC interactions are introduced. Spin polarization is in general small, for acceptable SOC values, but is amplified in nonhalf-filled systems and/or in the presence of weak bonds, leading in specific cases to spin polarization up to ∼ 10%. However, since sizable CISS effects are observed in several systems of a very different nature, we conclude that electron correlations in the simple Hubbard chain hardly explain CISS. In the process, we learned an important lesson: when SOC enters the model, the standard definition of the current operator in terms of the velocity dipole is no more adequate, since SOC itself enters the current operator.

The proposed approach lends itself quite naturally to address the role of nonadiabatic vibrational modes in correlated electron systems. Molecular vibrations modulating on-site energies, the Holstein modes, have no major effects. Peierls modes, modulating the hopping integrals, lead instead to more interesting physics. In the first place, Peierls modes, together with SOC, enter the definition of the current operator. As long as the current-carrying system is fully equilibrated in terms of both electronic and vibrational degrees of freedom, vibrational coupling only marginally affects CISS. However, if the vibrational coordinate is kept out of equilibrium in the current-carrying system, a very interesting result is obtained in terms of a finite spin polarization in the two-site Hubbard chain. In other terms, if vibrational modes are maintained out of equilibrium, nnn interactions are not needed to observe spin-polarization. This impressive result, in line with recent theoretical work,^53,61^ suggests that the chiral nature acquired by the ubiquitous vibrational degrees of freedom in chiral molecules can be the key to unravel the CISS physics.

## Supplementary Material





## Notes

Table illustrating the real space basis set; derivations of the spin–orbit coupling Hamiltonian and of the gauge transformations; additional computational results (PDF)
Transparent Peer Review report available (PDF)
