# Supplementary material for: Chirality-Induced Spin Selectivity: A Minimal Model
Source: J Phys Chem Lett. 2025 Aug 26;16(35):9107–15. doi: 10.1021/acs.jpclett.5c01813 (PMC12415877; doi:10.1021/acs.jpclett.5c01813)
Supplement: Supplementary file 1 [file jz5c01813_si_001.pdf]

Supporting Information

Chirality-induced spin selectivity: a minimal model

Lorenzo Savi,[†] Leonardo Celada,[‡] D.K. Andrea Phan Huu,[‡] Alessandro Chiesa,[‡] Stefano Carretta,[‡] and Anna Painelli[*,†]

†*Department of Chemistry, Life Science and Environmental Sustainability, University of Parma*

‡*Department of Mathematical, Physical and Computer Sciences University of Parma*

E-mail: anna.painelli@unipr.it

S1 The SOC term

The p orbital on site i can be written as a linear combination of the p orbitals with $l_z = \pm 1$:

$$|\psi_i\rangle = \frac{1}{\sqrt{2}} \left(p^i_{-1} e^{\mathrm{i}\theta i} - p^i_1 e^{-\mathrm{i}\theta i} \right) \tag{1}$$

where i is the imaginary unit. The one-electron matrix elements of the $\mathbf{l}_{ri} \cdot \mathbf{s}_r$ operator reads:

$$\langle \psi_k(r)\sigma'(r)| \, \mathbf{l}_{ri} \cdot \mathbf{s}_r \, |\psi_i(r)\sigma(r)\rangle = \langle \psi_k(r)| \, l_{z,ri} \, |\psi_i(r)\rangle \, \langle \sigma(r)| \, \hat{s}_z \, |\sigma(r)\rangle \, \delta_{\sigma,\sigma'} \tag{2}$$

$\sigma(r)$ is the spin function that describes electron r. Since the angular momentum of the electron is parallel to the z axis, the z component of the spin is conserved. Finally, in line with the choice made for the hopping integrals, we set the electronic matrix element proportional to the overlap between relevant orbitals, we get

$$\langle \psi_k(r)\sigma'(r)| \, \mathbf{l}_{ri} \cdot \mathbf{s}_r \, |\psi_i(r)\sigma(r)\rangle \propto \cos\theta \sin\theta \, \langle \sigma(r)| \, \hat{s}_z \, |\sigma(r)\rangle \, \delta_{\sigma,\sigma'} \delta_{k,i\pm 1} \tag{3}$$

and the SOC term in Eq. (2) reduces to Eq. (3).

S2 The real space basis

The basis set for the electronic calculation is defined by the configurations obtained assigning each electron to a specific site spin-orbital. We work on electronic subspaces defined by the configurations having the same S_z, as discussed in the main text. A bit-representation is adopted to store each configuration as a single integer number. Specifically, each site orbital is represented by a bit, whose value is either 0 or 1 to represent a void or an occupied site orbital. Table S1 shows the 36 basis states in the $S_z = 0$ subspace for a half filled 4-sites chain.

Table S1: The real-space basis set for a 4 sites 4 electrons system in the $S_z = 0$ subspace.

Integer representative number	bit representation	site representation
15	00 00 11 11	0 0 X X
27	00 01 10 11	0 β α X
30	00 01 11 10	0 β X α
39	00 10 01 11	0 α β X
45	00 10 11 01	0 α X β
51	00 11 00 11	0 X 0 X
54	00 11 01 10	0 X β α
57	00 11 10 01	0 X α β
60	00 11 11 00	0 X X 0
75	01 00 10 11	β 0 α X
78	01 00 11 10	β 0 X α
90	01 01 10 10	β β α α
99	01 10 00 11	β α 0 X
102	01 10 01 10	β α β α
105	01 10 10 01	β α α β
108	01 10 11 00	β α X 0
114	01 11 00 10	β X 0 α
120	01 11 10 00	β X α 0
135	10 00 01 11	α 0 β X
141	10 00 11 01	α 0 X β
147	10 01 00 11	α β 0 X
150	10 01 01 10	α β β α
153	10 01 10 01	α β α β
156	10 01 11 00	α β X 0
165	10 10 01 01	α α β β
177	10 11 00 01	α X 0 β
180	10 11 01 00	α X β 0
195	11 00 00 11	X 0 0 X
198	11 00 01 10	X 0 β α
201	11 00 10 01	X 0 α β
204	11 00 11 00	X 0 X 0
210	11 01 00 10	X β 0 α
216	11 01 10 00	X β α 0
225	11 10 00 01	X α 0 β
228	11 10 01 00	X α β 0
240	11 11 00 00	X X 0 0

Left column: The integer number representing each state. Central column: bit representation of the state. The spin-orbital on each site is represented by a bit in the order $1\alpha, 1\beta, 2\alpha, 2\beta, 3\alpha, 3\beta, 4\alpha, 4\beta$. 0 and 1 correspond to a void or occupied spin-orbital, respectively. Right column: site representation where X stands for doubly occupied sites, 0 for a void site and α, β indicate the spin of the electron in a single occupied site.

S3 Definition of the current operator

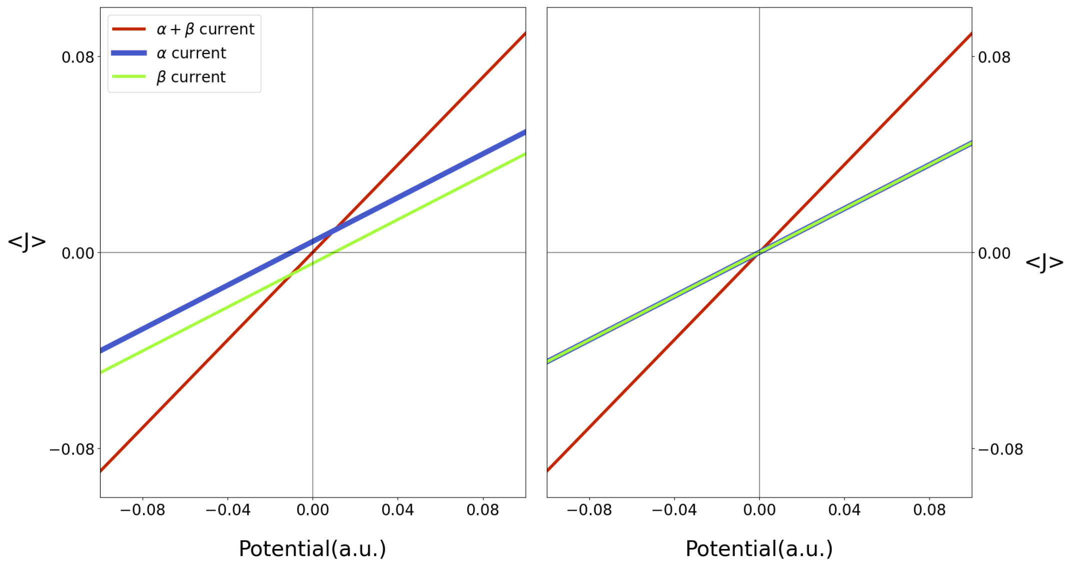

Figure S1: The two site Hubbard model with $t = 1$, $\chi = 0.01$, U=0, $\epsilon_1 = -\Delta$, $\epsilon_2 = \Delta$. Left: α- and β-spin current and the total current calculated setting the current operator proportional to the velocity operator, without accounting for the SOC contribution to the current operator. Right panel, the same quantities are calculated adopting the correct definition of the current, as defined by Eq. 12 on the main text. Finite α and β currents calculated at zero potential in the left panel signal the incorrect definition of the current operator.

S4 The gauge transformation for the Hubbard Hamiltonian with nearest-neighbor hopping

If nnn interactions are neglected the current-carrying Hamiltonian reads:

$$\begin{aligned} H(\lambda) &= \sum_{i=1}^{N} \epsilon_i \hat{n}_i + \sum_{i=1}^{N} U_i \hat{n}_{i\alpha}\hat{n}_{i\beta} \\ &\quad + \sum_{i=1}^{N-1} \left[-t_i \hat{b}_{ij} + \mathrm{i}\chi_i(\hat{v}_{i,i+1,\alpha} - \hat{v}_{i,i+1,\beta}) - \lambda_i \hat{j}_i^{i+1} \right] \end{aligned} \qquad (4)$$

where, for the sake of simplicity we have neglected the term accounting for intersite electrostatic interactions, that however does not affect the deiscussion below. The last line in

the above Hamiltonian describes all hopping terms. Focusing on just one of these terms we rewrite it as follows:

$$-t_i(1+\mathrm{i}\lambda_i)\mathbf{1}_{2\times 2}+\chi_i(\mathrm{i}-\lambda)\sigma_z = A_i\cdot\mathbf{1}_{2\times 2}+B_i\cdot\sigma_z \tag{5}$$

where the unit and the σ_z matrices are defined on the two dimensional spin subspace. The following transformation is defined on each site as follows:

$$\hat{U}_j = e^{\mathrm{i}j\phi_j\sigma_z} \tag{6}$$

since the transformation operators are unitary:

$$\hat{U}_j^\dagger\hat{U}_j=\hat{U}_j\hat{U}_j^\dagger=\mathbf{1}_{2\times 2}$$

we insert the identity in the Hamiltonian in Eq. 4:

$$\begin{aligned}\tilde{\mathbf{H}} &= \sum_{j=1}^{N}\sum_{\sigma}\epsilon_j c_{j,\sigma}^\dagger\hat{U}_j\hat{U}_j^\dagger c_{j,\sigma}+\sum_{j=1}^{N}U_j c_{j,\alpha}^\dagger\hat{U}_j\hat{U}_j^\dagger c_{j,\alpha}c_{j,\beta}^\dagger\hat{U}_j\hat{U}_j^\dagger c_{j,\beta}\\ &+\sum_{j=1}^{N-1}\sum_{\sigma}c_{j,\sigma}^\dagger\hat{U}_j\hat{U}_j^\dagger\left[-t_j(1+\mathrm{i}\lambda_j)+\chi_j(\mathrm{i}-\lambda_j)\sigma_z\right]\hat{U}_{j+1}\hat{U}_{j+1}^\dagger\ c_{j+1,\sigma}\mathrm{h.c.}\\ &=\sum_{j=1}^{N}\sum_{\sigma}\epsilon_j d_{j,\sigma}^\dagger d_{j,\sigma}+\sum_{j=1}^{N}U_j d_{j,\alpha}^\dagger d_{j,\alpha}d_{j,\beta}^\dagger d_{j,\beta}\\ &+\sum_{j=1}^{N-1}\sum_{\sigma}d_{j,\sigma}^\dagger\hat{U}_j^\dagger\left[-t_j(1+\mathrm{i}\lambda_j)+\chi_j(\mathrm{i}-\lambda_j)\sigma_z\right]\hat{U}_{j+1}\ d_{2,\sigma}+\mathrm{h.c.}\end{aligned}$$

Where:

$$d_{j,\sigma}=U_j^\dagger c_{j,\sigma}\qquad\qquad d_{j,\sigma}^\dagger=c_{j,\sigma}^\dagger U_j \tag{7}$$

We set:

$$\hat{U}_j^\dagger\left[-t_j(1+\mathrm{i}\lambda_j)+\chi_j(\mathrm{i}-\lambda_j)\sigma_z\right]\hat{U}_{j+1}=\left[A_j\cos(\phi_j)+\mathrm{i}B_j\sin(\phi_j)\right]\mathbf{1}_{2\times 2}+\left[B_j\cos(\phi_j)+\mathrm{i}A_j\sin(\phi_j)\right]\sigma_z$$

To gauge the soc term we impose:

$$\phi_j = \arctan\left(\frac{\chi_j}{t_j}\right)$$

while:

$$A_j\cos(\phi_j) + \mathrm{i}B_j\sin(\phi_j) = -\sqrt{t_j^2+\chi_j^2} - \mathrm{i}\lambda_j\frac{t_j^2-\chi_j^2}{\sqrt{t_j^2+\chi_j^2}}$$

So the final hamiltonian is:

$$\begin{aligned}\tilde{\mathbf{H}} = &\sum_{j=1}^{2}\sum_{\sigma=\uparrow,\downarrow}\epsilon_j d^\dagger_{j,\sigma}d_{j,\sigma} \; + \sum_{j=1}^{2}U_j d^\dagger_{j,\alpha}d_{j,\alpha}d^\dagger_{j,\beta}d_{j,\beta} \\ &- \left[\sqrt{t^2+\chi^2} + i\lambda\frac{t^2-\chi^2}{\sqrt{t^2+\chi^2}}\right]\sum_{\sigma=\uparrow,\downarrow} d^\dagger_{1,\sigma}d_{2,\sigma} + \text{ h.c.}\end{aligned} \tag{8}$$

The term SOC is completely gauged away setting:

$$\hat{U}_j = e^{\mathrm{i}j\arctan\left(\frac{\chi_j}{t_j}\right)\sigma_z} \tag{9}$$

Interestingly, the Lagrange multiplier does not appear in the transformation that holds true in the presence and in the absence of a running current. The Hamiltonian is no longer spin-dependent along $\hat{z}$ and therefore we cannot have any polarization along $\hat{z}$. Of course, the SOC cannot be gauged out if either nnn hopping or nnn SOC interactions are accounted for.

S5 Role of the connectivity

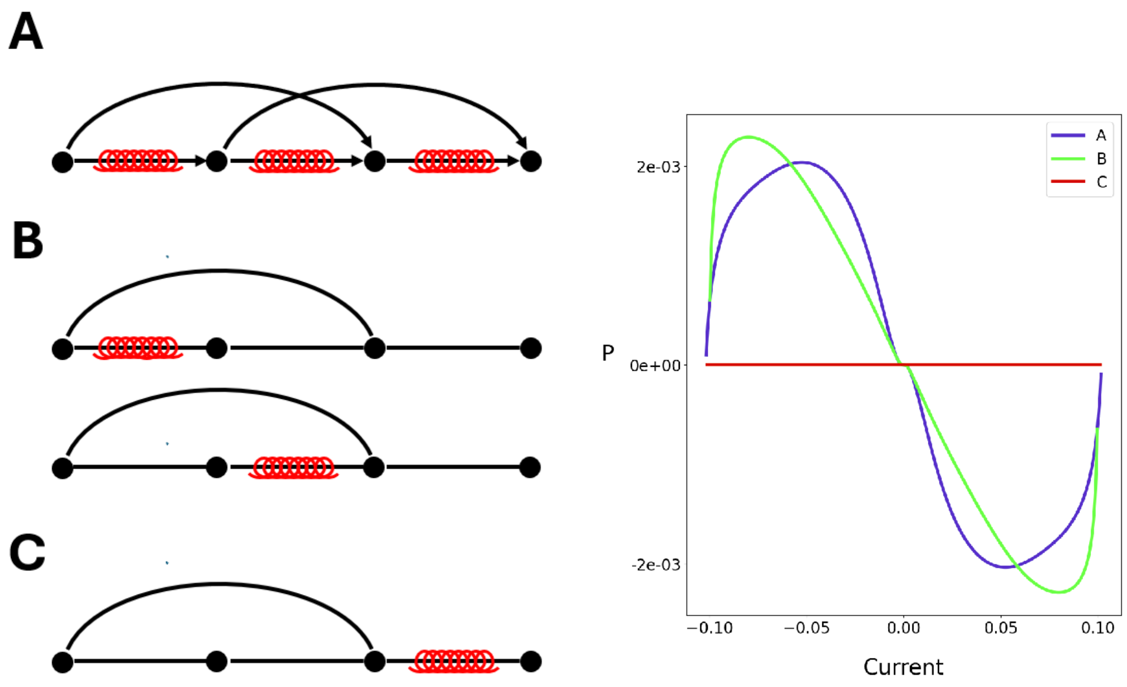

(a) (b)

Figure S2: Illustrative examples of the role of the connectivity of a 4 site chain. Left panel: a schematic view of the four site chain, where each black line refers to a hopping interaction and each red helix marks the SOC interaction. Right panel: the spin polarization as a function of current calculated for the different configurations displayed in the left panel, setting $t_{23} = 1$ as the energy unit, $t_{12} = t_{34} = 0.05$ and the nnn hopping t' =0.01. Moreover $\chi = 0.01$, $U = 2\Delta = 5$. The two systems marked as B lead to equivalent results.

S6 The half-filled 4-site Hubbard chain: additional results

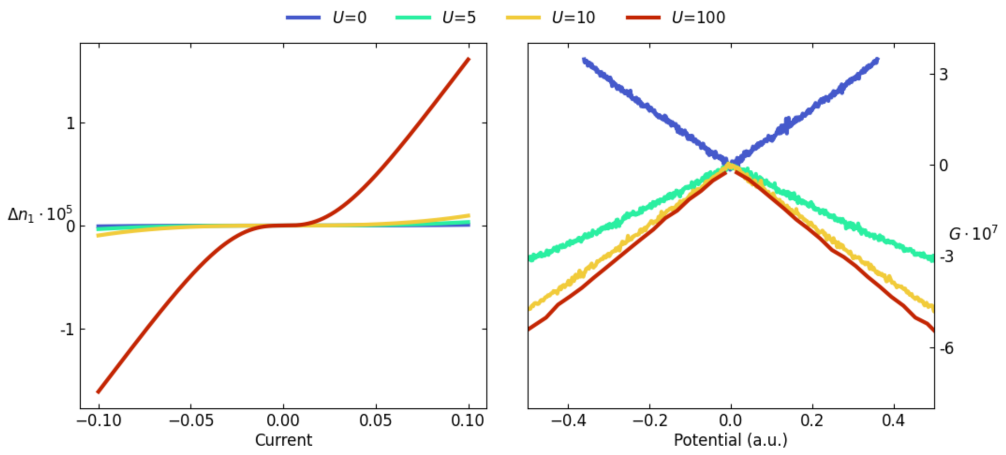

Figure S3: The 4 site Hubbard model with nn $t = 1$, and $\chi = 0.01$, the nnn $t' = 0.01$, and $\epsilon_i = 0$. The results are shown for different U, as per the legend. Left panel: the spin density on the first site (equal to the spin density on the last site) as a function of the current; right panel: the current anisotropy vs the applied potential.

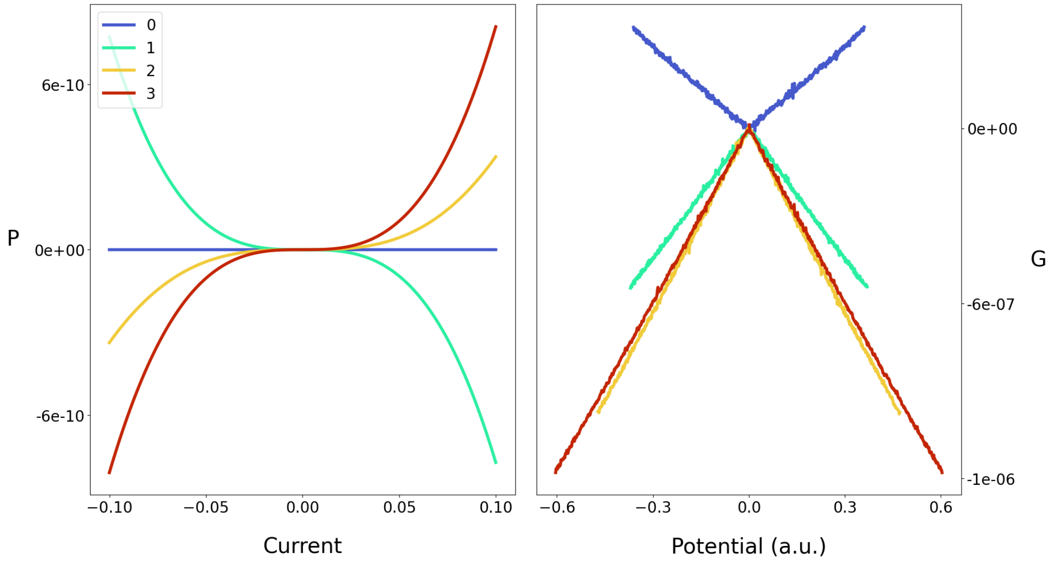

Figure S4: The 4 site Hubbard model with nn $t = 1$ and $\chi = 0.01$, nnn $t' = 0.01$, U=0. Results are shown for different Δ, as per the legend. Left panel: the polarization as a function of the current; right panel: the current anisotropy vs the applied potential.

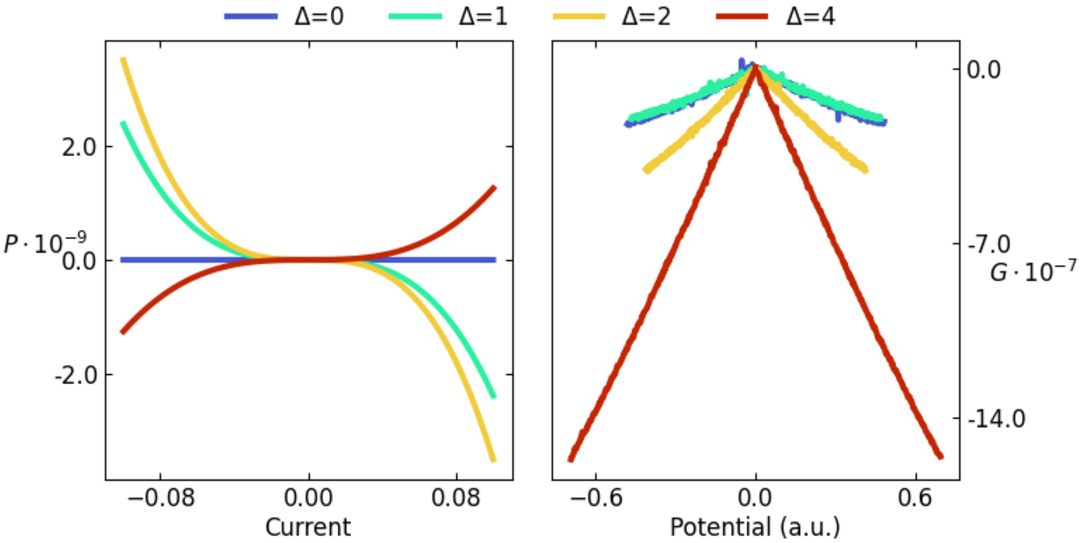

Figure S5: The 4 site Hubbard model with nn $t = 1$ and $\chi = 0.01$, nnn $t' = 0.01$, U=4. Results are shown fo different Δ, as per the legend. Left panel: the polarization as a function of the current; right panel: the current anisotropy vs the applied potential.

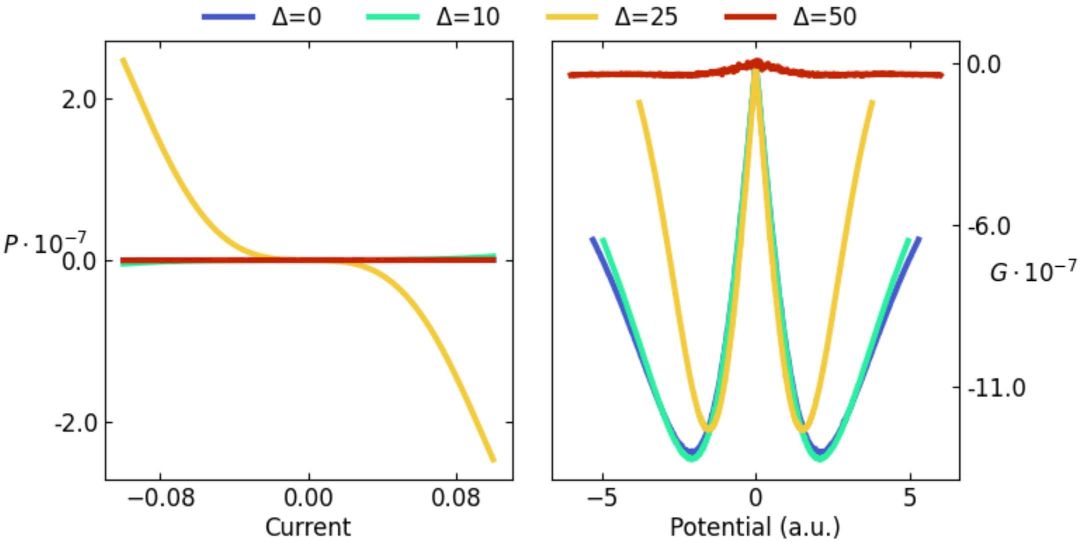

Figure S6: The 4 site Hubbard model with nn $t = 1$ and $\chi = 0.01$, nnn $t' = 0.01$, U=50. Results are shown fo different Δ, as per the legend. Left panel: the polarization as a function of the current; right panel: the current anisotropy vs the applied potential.

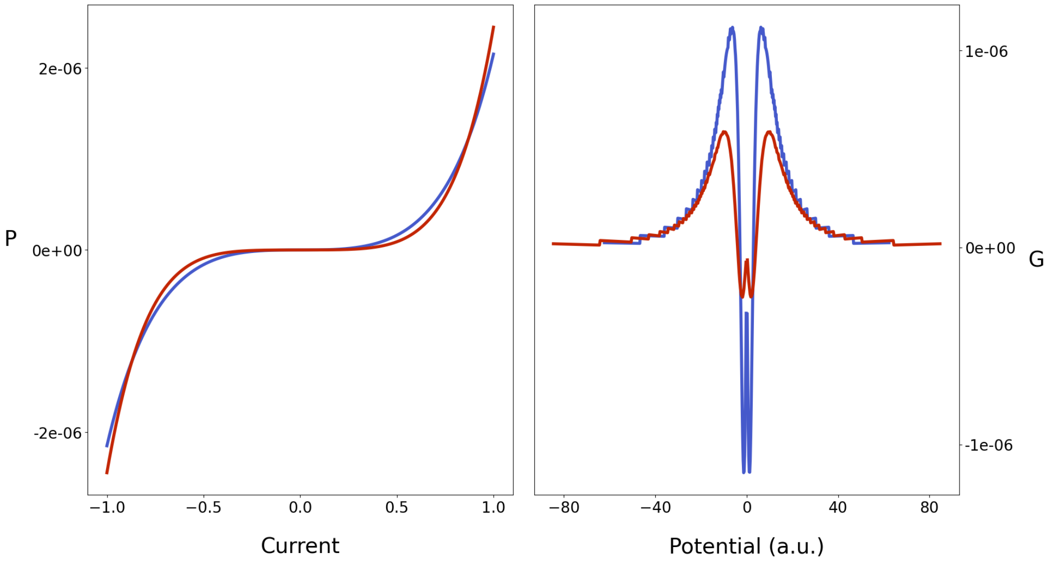

Figure S7: Intersite electrostatic interactions have a similar role as on-site energies. Result refer to a four sites Hubbard model with nn hopping $t = 1$ and $\chi = 0.01$, nnn hopping $t' = 0.01$ and U=1. Blue lines show results for a system with $W_{ij} = 0$ and $2\Delta = 1$; red lines show results for a system with nearest neighbor $W = 1.5$ and $2\Delta = 0$. Left panel shows the evolution of the spin polarization with the current, the right panel shows the current anisotropy vs the applied voltage.

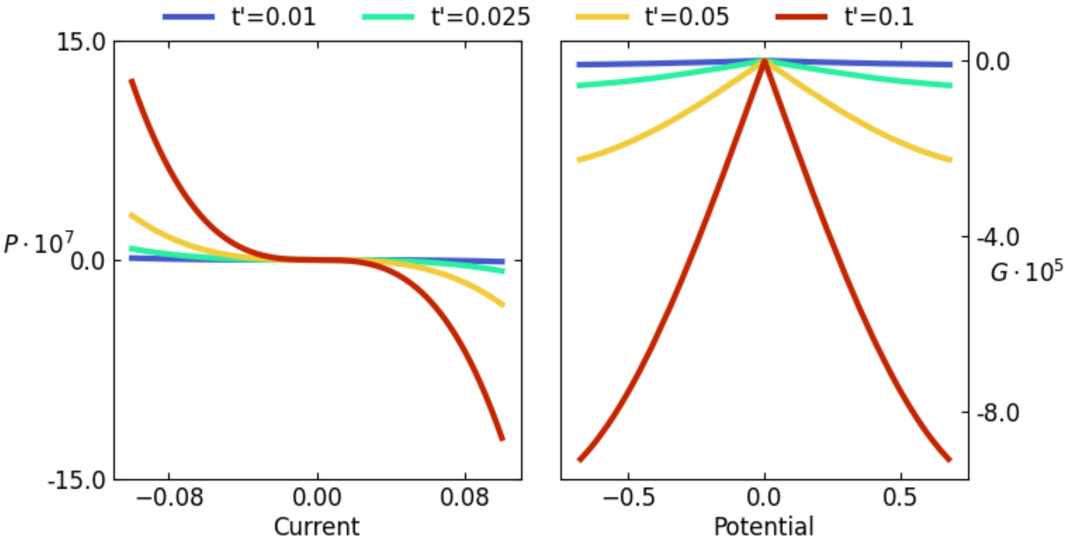

Figure S8: The 4 site Hubbard model with nn $t = 1$ and $\chi = 0.01$, U=10, $2\Delta = 5$. Results are shown for different nnn $t' =$, as per the legend. Left panel: the polarization as a function of the current; right panel: the current anisotropy vs the applied potential.

S7 The non-half-filled Hubbard chain

The real space approach applies irrespective of the filling. Here we discuss results obtained for a 4-site Hubbard chain with 2 or 6 electrons. The first observation is that either non-nearest neighbor interactions or inequivalent on-site energies break electron-hole symmetry, so that results obtained for 4 or 6 electrons are not equivalent. Fig. S9 shows results for a non-correlated chain and all equivalent sites. Since the polarization vanishes in these conditions, the left panel shows the spin unbalance on site 1 (equal to the one on site 4 so that the polarization vanishes). The effect of the number of electron on the spin unbalance is marginal, while the current anisotropy increseas by two orders of magnitude when going from 4 to either 2 or 6 electrons. Fig.S10 shows results for a correlated systems system with nn $t = 1$ and $\chi = 0.01$, nnn $t' = 0.01$, $U = 2\Delta$=10, In this case, both the spin polarization and the current anisotropy a two-order-of-magnitude amplification with respect to the half-filled case.

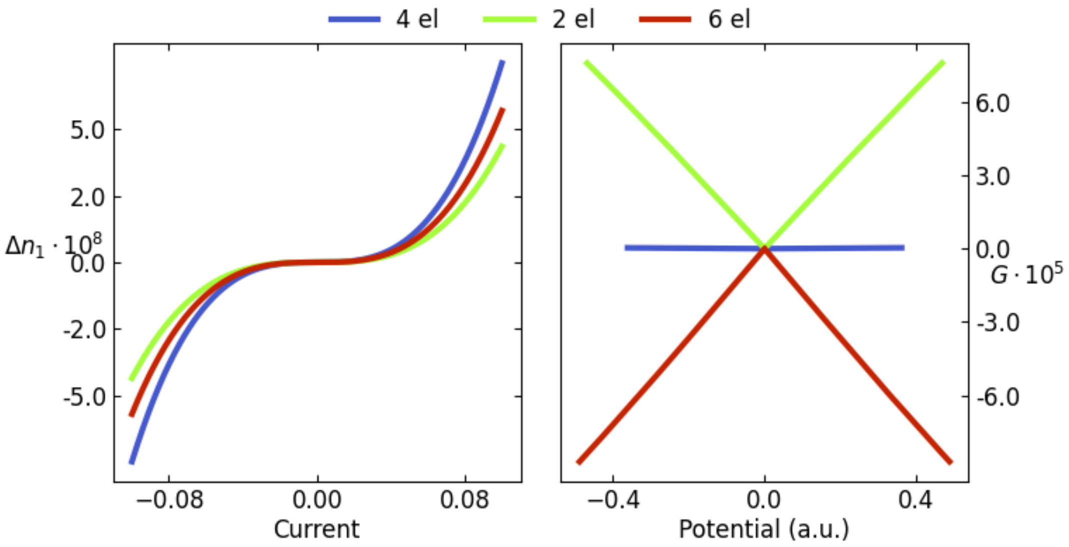

Figure S9: The 4 site Hubbard chain with $\epsilon_i = U = 0$, nn $t = 1$ and $\chi = 0.01$, nnn $t' = 0.01$. Blu lines: half filled systems. Green and red lines refer to the system with 2 and 6 electrons,respectively. Left panel: the spin unbalance on site 1 as a function of the current; right panel: the current anisotropy vs the applied potential.

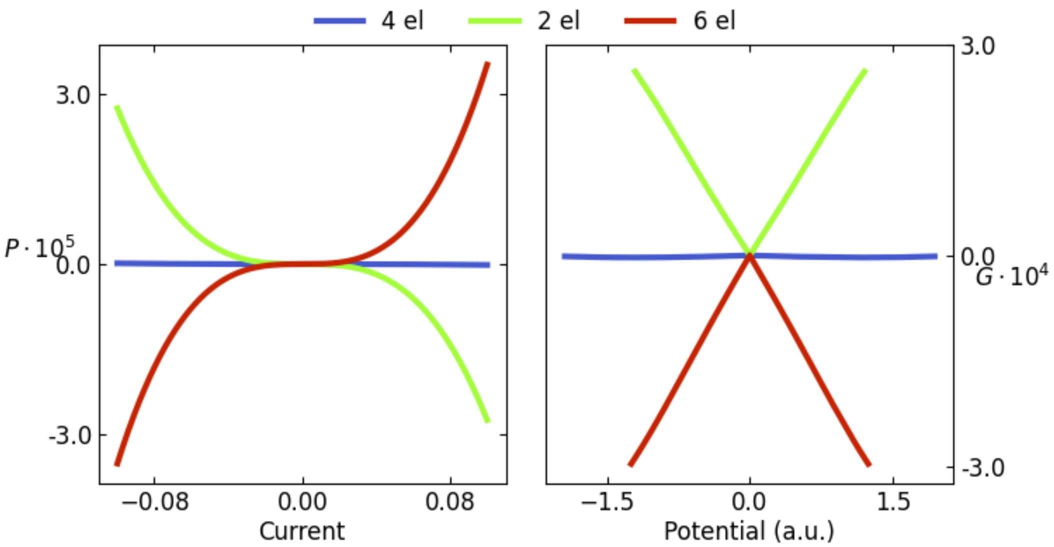

Figure S10: The 4 site half-filled Hubbard chain with $U = 2\Delta = 10$, nn $t = 1$ and $\chi = 0.01$, nnn $t' = 0.01$. Blu lines: half filled chain. Green and red lines: 2 and 6 electrons, respectively. Left panel: the polarization as a function of the current; right panel: the current anisotropy vs the applied potential.

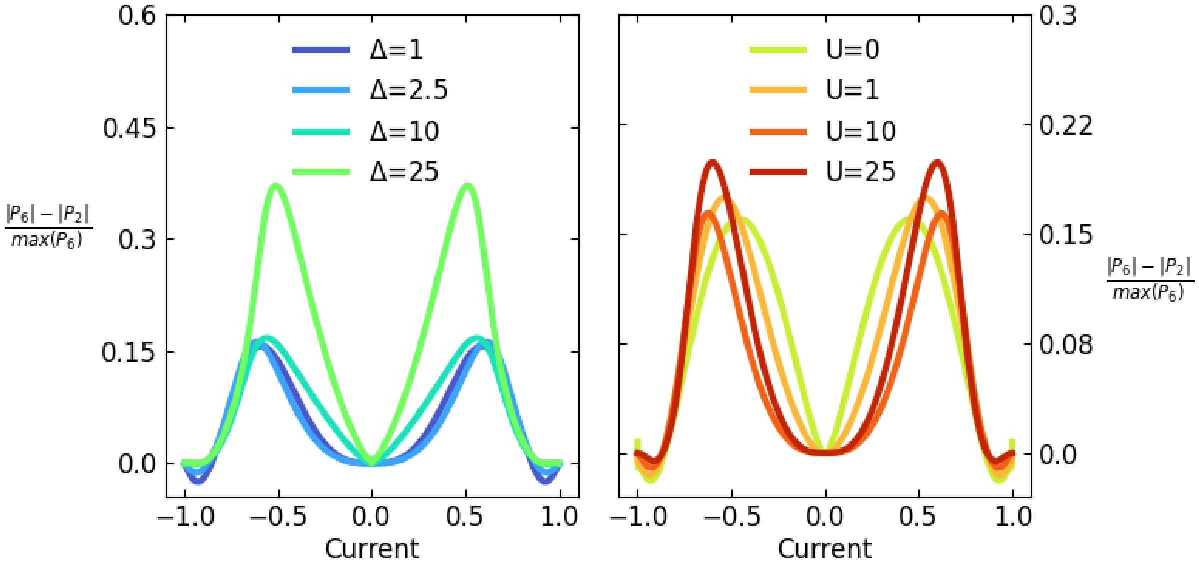

Figure S11: The 4 site Hubbard chain with nn $t = 1$ and $\chi = 0.01$, nnn $t' = 0.01$. Both panels display the difference in spin polarization between the 6-electron case and the 2-electron case, normalized for the maximum of the 6-electron case. Left: U=5, Δ varies as per the legend.Right: Δ=2.5, U varies as per the legend.

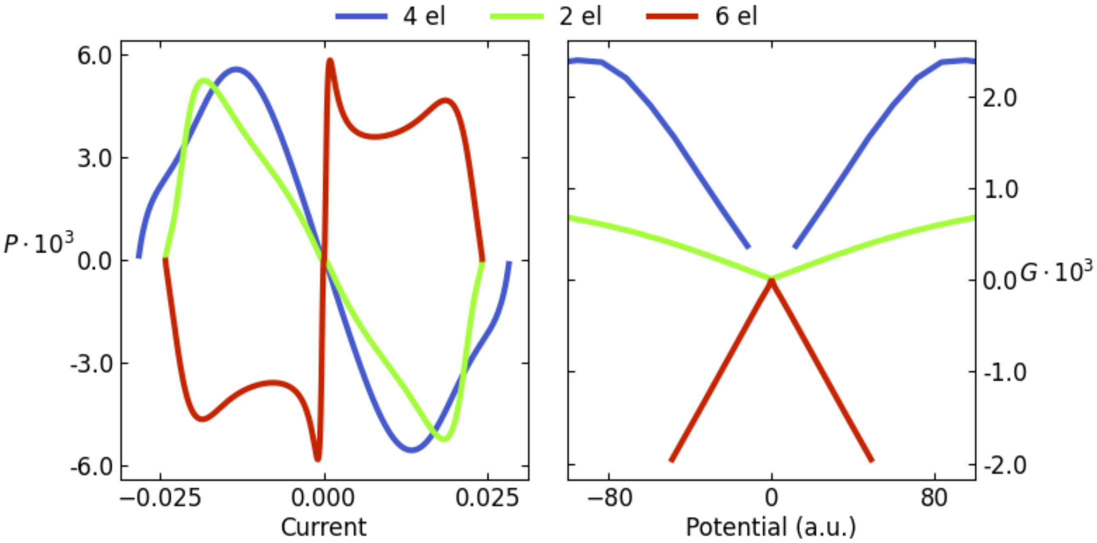

Figure S12: The 4 site half-filled Hubbard chain with $2\Delta = U = 5$, $t_{12} = t_{34} = 1$, $t_{23} = 0.01$, nn $\chi = 0.01$ and nnn $t' = 0.01$. Blu lines: half filled chain. Green and red lines: 2 and 6 electrons, respectively. Left panel: the polarization as a function of the current; right panel: the current anisotropy vs the applied potential.

S8 Introducing Holstein phonons

A Holstein vibration is introduced on each site, which modulates the on-site energy, so that the nn Hamiltonian in Eq. 4 reads:

$$H(\lambda) = \sum_{j=1}^{N} \sum_{\sigma} c^\dagger_{j,\sigma} c_{j,\sigma} \left[\epsilon_j + g_j (a^\dagger_j + a_j) \right] + \sum_{j=1}^{N} U_j c^\dagger_{j,\alpha} c_{j,\alpha} c^\dagger_{j,\beta} c_{j,\beta} \tag{10}$$

$$+ \sum_{j=1}^{N} \hbar\omega_j \left(a^\dagger_j a_j + \frac{1}{2} \right) + \sum_{\sigma} c^\dagger_{1,\sigma} \left[-t + i\chi\sigma_z \right] c_{2,\sigma} + \text{h.c.} \tag{11}$$

$$-\lambda \left[it \left(\sum_{\sigma=\uparrow,\downarrow} c^\dagger_{1,\sigma} c_{2,\sigma} + \text{h.c.} \right) - \chi \left(\sum_{\sigma=\uparrow,\downarrow} c^\dagger_{1,\sigma} \sigma_z c_{2,\sigma} + \text{h.c.} \right) \right] \tag{12}$$

We apply the Lang-Firsov transformation with the following ansatz:

$$\mathbf{S_j} = \sum_{\sigma} \frac{g_j}{\hbar\omega_j} c^\dagger_{j,\sigma} c_{j,\sigma} \left(a^\dagger_j - a_j \right) \tag{13}$$

From $\mathbf{S_j}^\dagger = -\mathbf{S_j}$, it follows $e^{\mathbf{S_j}} = \left(e^{-\mathbf{S}_j}\right)^\dagger$. Applying the unitary transformation $\tilde{\mathbf{H}} = e^{\mathbf{S_j}}\mathbf{H}e^{-\mathbf{S_j}}$, the transformed operators on site j read:

$$\tilde{a}_j = a_j - \sum_{\sigma=\uparrow,\downarrow} \frac{g_j}{\hbar\omega_{0,j}} c^\dagger_{j,\sigma} c_{j,\sigma} \qquad \tilde{a}^\dagger_j = a_j - \sum_{\sigma=\uparrow,\downarrow} \frac{g_j}{\hbar\omega_{0,j}} c^\dagger_{j,\sigma} c_{j,\sigma} \tag{14}$$

$$\tilde{c}_{j,\sigma} = c_{j,\sigma}\, e^{-\frac{g_j}{\hbar\omega_{0,j}}\,(a^\dagger_j - a_j)} \qquad \tilde{c}^\dagger_{j,\sigma} = c^\dagger_{j,\sigma} e^{+\frac{g_j}{\hbar\omega_{0,j}}\,(a^\dagger_j - a_j)} \tag{15}$$

while, for site $i \neq j$ the operators stay unchanged:

$$\tilde{c}_{i,\sigma'} = c_{i,\sigma'}$$

$$\tilde{a}_i = a_i$$

Repeating the transformation on each site, we end up with the transformed operator:

$$\tilde{c}^\dagger_{j,\sigma}\tilde{c}_{j,\sigma} = c^\dagger_{j,\sigma} c_{j,\sigma}$$

$$\tilde{c}^\dagger_{j,\sigma}\tilde{c}_{j',\sigma} = c^\dagger_{j,\sigma} c_{j',\sigma}\ \exp\left\{\frac{g_j}{\hbar\omega_{0,j}}\,(a^\dagger_j - a_j)\ -\ \frac{g_{j'}}{\hbar\omega_{0,j'}}\,(a^\dagger_{j'} - a_{j'})\right\} = c^\dagger_{j,\sigma} c_{j',\sigma}\Lambda_{j,j'}$$

$$\tilde{a}^\dagger_j + \tilde{a}_j = a^\dagger_j + a_j - 2\sum_{j=1}^{2}\sum_{\sigma=\uparrow,\downarrow} \frac{g_j}{\hbar\omega_0}\, c^\dagger_{j,\sigma} c_{j,\sigma}$$

$$\tilde{a}^\dagger_j\tilde{a}_j + \frac{1}{2} = a^\dagger_j a_j + \frac{1}{2} - \sum_{j=1}^{2}\sum_{\sigma=\uparrow,\downarrow} \frac{g_j}{\hbar\omega_j}\, c^\dagger_{j,\sigma} c_{j,\sigma}(a^\dagger_j + a_j) + \left[\sum_{j=1}^{2}\sum_{\sigma=\uparrow,\downarrow} \frac{g_j}{\hbar\omega_0}\, c^\dagger_{j,\sigma} c_{j,\sigma}\right]^2$$

Finally, the trasformed Hamiltonain is:

$$\begin{aligned}\tilde{\mathbf{H}} = &\sum_{j=1}^{N}\sum_{\sigma}\left[\epsilon_j - \frac{g_j^2}{\hbar\omega_j}\right] c^\dagger_{j,\sigma} c_{j,\sigma}\ +\ \sum_{j=1}^{N}\hbar\omega_j\left(a^\dagger_j a_j + \frac{1}{2}\right) + \sum_{j=1}^{2}\left(U_j - \frac{2g_j^2}{\hbar\omega_j}\right) c^\dagger_{j,\alpha} c_{j,\alpha} c^\dagger_{j,\beta} c_{j,\beta} \\ &+ \sum_{j=1}^{N-1}\sum_{\sigma}\left[-t_j(1+\mathrm{i}\lambda) + \chi_j(\mathrm{i}-\lambda)\sigma_z\right] c^\dagger_{j,\sigma} c_{i+1,\sigma}\Lambda_{i,i+1} +\ \text{h.c.}\end{aligned} \tag{16}$$

Apart from a renormalization of the on-site energies and of the on-site electron-electron

repulsion, the electronic part of the Hamiltonian has the same form as the purely electronic Hamiltonain in Eq. 4. Accordingly, as long as only nn interactions are accounted for, the same gauge transformation can be applied as discussed in Section S4, proving that Holstein phonons cannot lead to spin polarization in a system with only nn hopping terms.

S9 Introducing Peierls phonons

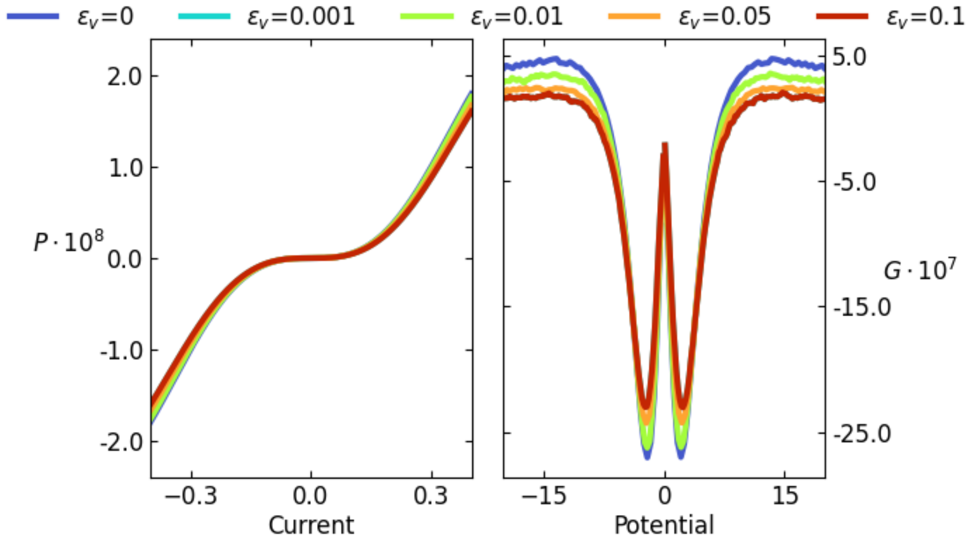

Figure S13: The same as Fig. 3a (main text) but with $U = 0$.

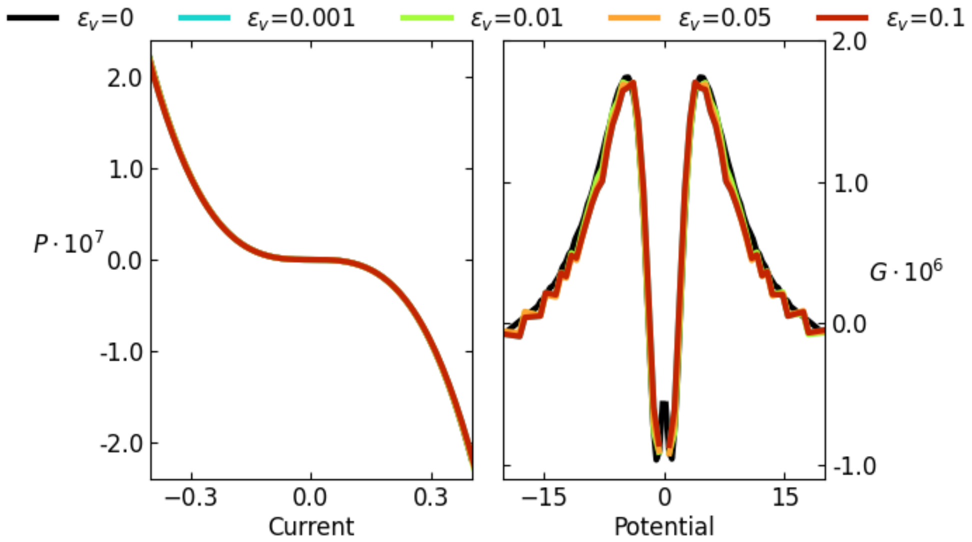

Figure S14: The 4-sites Hubbard chain with nn $t = 1$ and $\chi = 0.01$, nnn $t' = 0.01$, $U = 2\Delta = 5$, a vibrational mode is coupled in an anti-symmetric way to the bond between site 1 and 2 and sites 3 and 4 with interaction term: $g_\nu(\hat{a}_\nu^\dagger + \hat{a}_\nu)(\hat{b}_{12} - \hat{b}_{34})$ with $\omega = 0.1$ and different ϵ_v, as per the legend.

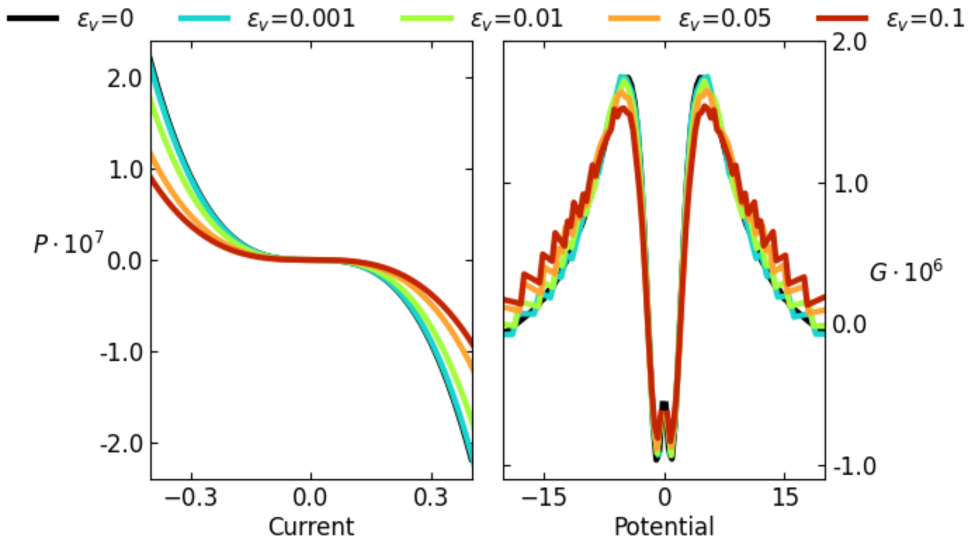

Figure S15: The same as Fig. S14 but for symmetric coupling: $g_\nu(\hat{a}_\nu^\dagger + \hat{a}_\nu)(\hat{b}_{12} + \hat{b}_{34})$.

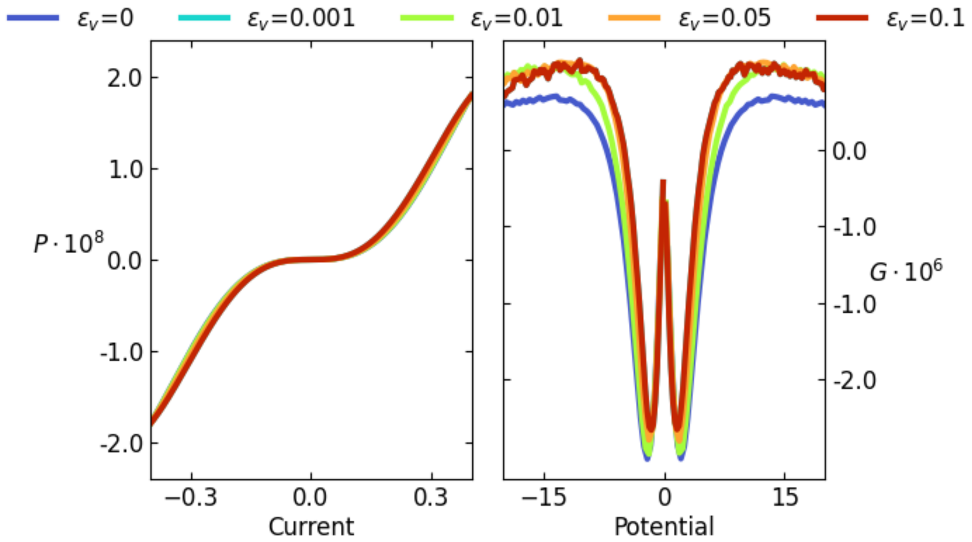

Figure S16: The same as Fig. S14 but for U=0.

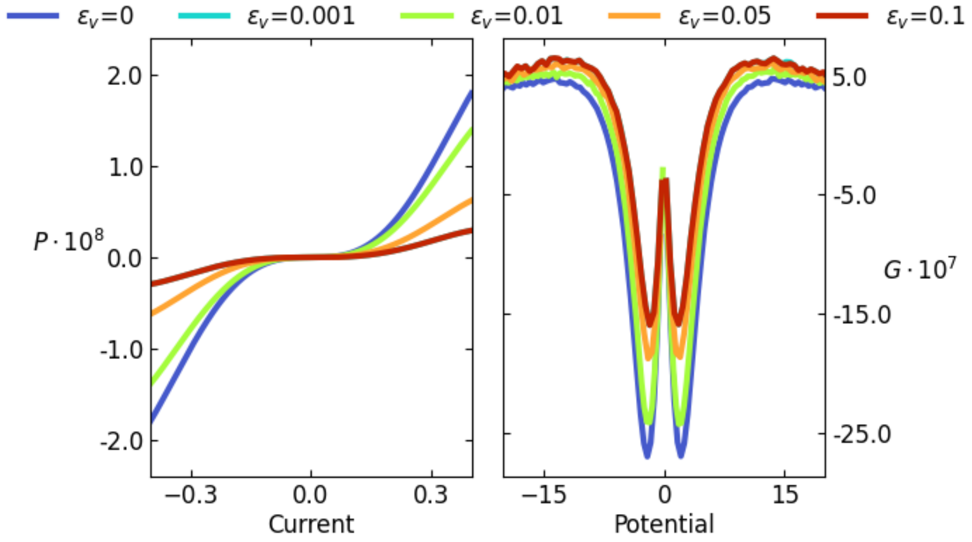

Figure S17: The same as Fig. S15 but for U=0.

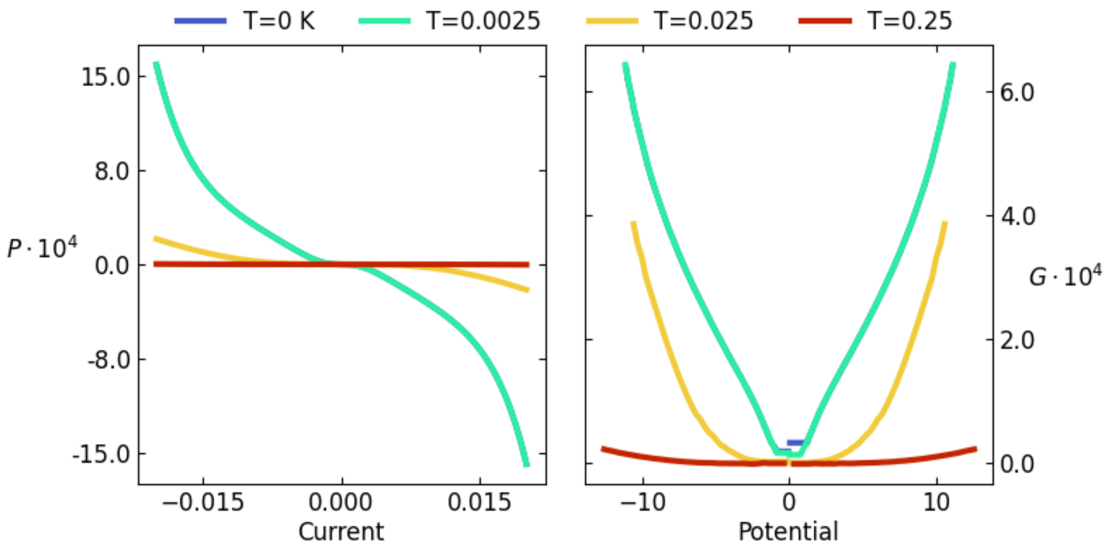

Figure S18: The 4 site Hubbard model with $t_{23} = 1$,$t_{12} = t_{23} = 0.05$ and $\chi = 0.01$, nnn $t' = 0.01$, $U = 2\Delta = 5$,, a vibrational mode is coupled to bond between sitew 1 and 2 and sites 3 and 4 in an anti-symmetric way is with $\omega = 0.1$ and $\epsilon_v = 0.01$. Results are shwon for different temperatures, as per the legend.

S10 CD spectra

The calculation of CD spectra in isotropic samples requires the calculation of the rotational strength $R(\omega)$:

$$R(\omega) \propto \sum_f \frac{Im\{\vec{\mu}_{gf} \cdot \vec{m}_{fg}\}}{\omega - \omega_{fg}} \tag{17}$$

where the sum runs over the excited states, $\vec{\mu}_{gf}$ and $\vec{m}_{fg}$ are the transition dipole moment and the magnetic dipole moment between the ground g and the f state (the expression holds in the hypothesis that only the ground state is populated, extending the expression to finite temperature is trivial). In the adopted model, the electric and magnetic dipole moments are collinear and are directed along the z axis. Specifically, in the real space basis the dipole moment operator is diagonal and reads:

$$\hat{\mu}_z = \sum_j (Z_j - \hat{n}_j) j \tag{18}$$

In the same basis, the magnetic dipole moment is:

$$\hat{m}_z \propto \mathrm{i}[\hat{r}, \hat{H}] = i \sum_{j<k,\sigma} t_{jk} \hat{c}^{\dagger}_{j\sigma} \hat{c}_{k\sigma} + h.c. \tag{19}$$

After having diagonalized the Hamiltonian, relevant transition elements are calculated and the CD spectra are then plotted.
