# Supplementary material for: Chirality-Induced Spin Selectivity: A Minimal Model
Source: J Phys Chem Lett. 2025 Aug 26;16(35):9107–15. doi: 10.1021/acs.jpclett.5c01813 (PMC12415877; doi:10.1021/acs.jpclett.5c01813)
Supplement: Supplementary file 2 [file jz5c01813_si_002.pdf]

jz-2025-01813t.R1

Name: Peer Review Information for "Chirality induced spin selectivity: a minimal model"

First Round of Reviewer Comments

Reviewer: 1

Comments to the Author

This paper presents a theoretical study aimed at elucidating the CISS effect using a simplified model incorporating electron correlation and molecular vibrations. In the first part, the authors demonstrate that nnn hopping or spin–orbit coupling (SOC) interactions are necessary in the chiral one-dimensional Hubbard model. Doping enhances spin selectivity by up to 10%. In the second part, the authors introduce a non-adiabatic vibrational mode, which appears to generate a quasi-particle accompanying the moving electrons. In this case, nnn hopping is no longer required, and the nonequilibrium state of the vibration is sufficient to produce the CISS effect.

The study is interesting for its inclusion of both electron correlation and molecular vibration in a minimal model—two aspects that are increasingly recognized as essential elements in microscopic models of the CISS effect.

I have only a few comments for the authors to consider.

1. The introduction is too lengthy for a letter format. In my opinion, the first two paragraphs could be reduced by half, although the references to previous work are informative.

2. The model using twisting p orbitals has been previously employed by the Akera group. For instance, see Phys. Rev. B 104, 035431 (2021) and J. Chem. Phys. 159, 074701 (2023). These references should be cited.

3. Lines 27–31 on page 8 are redundant and could be trimmed.

4. Regarding the absence of spin-flip processes in the model (e.g., page 10), it would be helpful to discuss how this relates to the spin filter and spin polarizer models of the CISS effect.

In summary, after addressing the comments above, I would be happy to recommend this manuscript for publication in JPCL.

Reviewer: 2

Comments to the Author

In this manuscript, the authors discuss a theoretical model that has, to the best of my knowledge, not been applied to chiral induced spin selectivity (CISS) so far, based on a Lagrangian multiplier approach for enforcing electron currents through molecules under steady-state conditions. As a result, it is found that vibrations play an important role in CISS, as has been suggested in several other theoretical reports on CISS, employing other models. It is interesting to see what this particular model has to say on the subject, and the analysis is well done and the results are thoroughly discussed. At the same time, it is not quite clear which new experimentally relevant insights this model can add. Based on the questions and comments below, it is not quite clear to me whether the publication criteria for JPCL are fulfilled.

1) The model describes a linear chain, in which chirality is introduced by twisting p orbitals. Such electronic, as opposed to structural, chirality, is a fascinating topic, which has been studied, among others, by Solomon, Garner, and Corminboeuf. I would suggest acknowledging this in the manuscript.

2) Experimental research on CISS so far has focused on structurally chiral systems. Why was a model system chosen here where chirality is only present on the electronic level, and what is the intended relation with the experiment?

3) It is interesting to see what happens in connection with CISS when employing this particular model. It still remains unclear what this model can add to understanding the experiment that other previously studied models cannot.

4) In typical CISS experiments, the variation of polarization with bias voltage (which controls current) is relatively small (see, e.g., https://doi.org/10.1002/smll.202308233), at least compared to the strong increase with current seen here (e.g. Fig 1a). This should be discussed in comparison with the experiment.

5) The finding that moving away from half filling and weak bonds promote polarization are interesting, but how do they compare with existing experimental data?

Overall, it appears that a lot of interesting things can be learned from this work on the behavior of this model, but it is less clear what can be learned for the experiment and for understanding CISS.

The language appears a bit odd in places ("renormalize way") amd has occasional grammar issues - it may make sense to proofread the manuscript accordingly.

Reviewer: 3

Comments to the Author

Report on jz-2025-01813t

Just like the authors point out, the CISS effect is an intriguing phenomenon which is under much debate as to how to understand the origin of the effect. Whether it is poorly understood may be a question of whether there exists any quantitatively predictable theoretical model for the phenomenon or not. I would certainly agree that there is yet no quantitive theory that provides the answers for any system, molecular or solid state, that has been used in experiments. On the other hand, there is a fair bit of qualitative understanding that one should not simply dismiss. Nonetheless, the CISS appears to be difficult to formulate in terms of simple effective models.

This is the quest of the authors to the manuscript at hand and one can say that, despite some bit of success they fail in most ways others do in convincingly reproducing the CISS

effect. At least when looking the numbers obtained through the calculations. I understand the vertical scales on the figures as 10^-6, 10^-4, and 10^-1 for the current anisotropy. By the way, current anisotropy is a good to use in the context of transport since, and I agree with the authors, the spin-polarization is not measured in this set-up. For the photoemission measurements, on the other hand, it is the spin-polarization that is measured which is why the terms makes sense in this set-up. Nonetheless, 10 % of current anisotropy is a good number.

Since the numbers do not match with experiments very well, except for some special conditions, which does not satisfy the authors – I praise this honesty, something which is not common these days – raises the question of whether there is physical insight enough to warrant publication of the results. On the other hand, we cannot blindly stare at the numbers. We should also be aware that negative results are quite important and for this reason, however, not only, I suggest that the paper should be published. A second reason is that the authors propose an alternative approach to the problem in which both direct Coulomb interactions and coupling to nuclear vibrations are included, yet allowing for simulations of non-equilibrium conditions. This is valuable and gives good cause for exploring the CISS effect using this approach.

Because, electron interactions would necessarily be part of the mechanism behind the CISS effect. There is nothing else in the set-up that can generate an anisotropy of the kind that is repeatedly measured by various groups using a wide range of conditions and a wide range of chiral molecules. Hence, although this paper is not the first attempt in which electron correlations are incorporated in the modeling, it is an important contribution and builds in the direction in which further research should be going.

Nonetheless, there are a few issues that the authors should address before the paper can be published.

1) In the abstract, and also elsewhere in the paper, the authors mention that the CISS effect cannot be obtained in a two site electronic system. This would have to be a trivial statement unless I am missing something. At least four sites are necessary for the construction of a chiral chain of sites. What is the intention with stating the, what I perceive as a, trivial and obvious fact?

2) In the literature, as well as in the present paper, the fact that the spin-orbit coupling is small is perceived as a problem concerning the spin-polarization and current anisotropy. However, there is no contradiction in this relation since spin-orbit coupling cannot on its own spin-polarize, it can only mix states. On the one hand, spin-orbit coupling is necessary since it couples the structure to the electronic spin. On the other hand, however, since the spin-orbit coupling typically is included at the single-electron level, and since it is not magnetic field-like, the result is a mixing of states, nothing else. In addition, interactions may enhance the effect of the spin-orbit coupling. I think the author should discuss this misunderstanding in the field and also comment on that the spin anisotropy has to originate from interactions in one or another way.

3) The authors neglect the spin-flip part of the spin-orbit coupling, something which is a trouble. By referring to a paper, which is also already old in this context, where this was a result of simulations, the authors take for granted that this assumption is safe. I disagree, both by that it is an incorrect approach to clarify the physics of a phenomenon, that with the author's words is poorly understood, and also since there is nothing ad hoc that suggests the correctness of such omission. Even so, DFT simulations cannot be used as telling the right answers to a question that, thus far, cannot be addressed using DFT. In fact, it was discussed in a recent paper [J Phys Chem Lett, 16, 4346 (2025)] that the transverse spin components, hence, spin-flip mechanisms are not negligible at all. I think that the authors should reconsider their simulations with the transverse spin-orbit coupling components included as well. Physically, one should expect the spins in the structure to be non-collinear and, therefore, reducing to a collinear approach is just not good enough. It can also be argued on good grounds that the CISS effect has to do with transfer of angular momentum within the structure, something that seems to go missing with the current assumptions.

Just below the assumption of collinear spin-orbit coupling, there is a mention of the S_z subspace. It is not clear what S_z refers to, although I interpret it as being related to the total spin of the molecule.

4) The authors claim that spin-orbit coupling between next nearest sites is sufficient to generate spin-polarization. However, spin-orbit coupling can by itself not generate spin-polarization, it has to be presence of the electron interactions that makes the difference.

Overall, I urge the authors to emphasize the crucial role interactions have for a meaningful modeling of the system to address the CISS effect.

In summary, the paper should be published provided that the authors address the questions raised above in a serious way.

Author's Response to Peer Review Comments:

Reply to Referees

We thank all reviewers for taking time to read the manuscript and for the general appreciation of our work. The reviewer’s comments are pertinent and useful, and offer us a valuable opportunity to improve the presentation.

We have revised the manuscript accordingly and we are confident that in the present form the manuscript meets standard for publication in JPCL.

For clarity, referees’ comments are reported in blue, our answer in black.

Reviewer: 1

Recommendation: This paper is publishable subject to minor revisions noted. Further review is not needed.

Comments:

This paper presents a theoretical study aimed at elucidating the CISS effect using a simplified model incorporating electron correlation and molecular vibrations. In the first part, the authors demonstrate that nnn hopping or spin–orbit coupling (SOC) interactions are necessary in the chiral one-dimensional Hubbard model. Doping enhances spin selectivity by up to 10%. In the second part, the authors introduce a non-adiabatic vibrational mode, which appears to generate a quasi-particle accompanying the moving electrons. In this case, nnn hopping is no longer required, and the nonequilibrium state of the vibration is sufficient to produce the CISS effect. The study is interesting for its inclusion of both electron correlation and molecular vibration in a minimal model—two aspects that are increasingly recognized as essential elements in microscopic models of the CISS effect.

We thank the reviewer for the very positive evaluation of our work

I have only a few comments for the authors to consider.

1. The introduction is too lengthy for a letter format. In my opinion, the first two paragraphs could be reduced by half, although the references to previous work are informative.

We have dropped several sentences in the introduction, that were indeed redundant.

2. The model using twisting p orbitals has been previously employed by the Akera group. For instance, see Phys. Rev. B 104, 035431 (2021) and J. Chem. Phys. 159, 074701 (2023). These references should be cited.

The model from Akera is more elaborate than our model: it considers atomic sites in a helical geometry and accounts for s and p onsite orbitals. Indeed, the suggested relevance of LS coupling in CISS is very interesting and we added a line in the text and the two references (ref 26 and 27).

3. Lines 27–31 on page 8 are redundant and could be trimmed.

We dropped a few (redundant) lines

4. Regarding the absence of spin-flip processes in the model (e.g., page 10), it would be helpful to discuss how this relates to the spin filter and spin polarizer models of the CISS effect.

This is an interesting observation. Indeed, the absence of spin-flip is inherent in our model, since, by construction, only the z component of the angular momentum enters into play. Several CISS models have been proposed where spin-flip terms are disregarded (e.g. refs 19, 21 53 and 62 in our manuscript), and some authors claim that this is a good approximation. That said, the possible role of spin-flip terms is a very interesting and urgent research topic. We are currently working on a more elaborate model, where, arranging the atoms in a truly 3D chiral structure, we can account for all SOC terms, including those responsible for spin-flips. This issue is shortly addressed in the manuscript: "*In the adopted model, the electron orbital momentum is aligned along the chain and the SOC interaction necessarily preserves the z component of the total spin, ˆ Sz, so that spin-flips are not accounted*

for. This is an inherent feature of the proposed model, but investigating the role of spin flips in CISS would definitely be interesting, particularly in view of the contrasting opinions in recent literature.16,42"

In summary, after addressing the comments above, I would be happy to recommend this manuscript for publication in JPCL.

Additional Questions:

Urgency: High

Significance: High

Novelty: High

Scholarly Presentation: High

Is the paper likely to interest a substantial number of physical chemists, not just specialists working in the authors' area of research?: Yes

Reviewer: 2

Recommendation: Reconsider as an article in The Journal of Physical Chemistry A/B/C.

Comments:

In this manuscript, the authors discuss a theoretical model that has, to the best of my knowledge, not been applied to chiral induced spin selectivity (CISS) so far, based on a Lagrangian multiplier approach for enforcing electron currents through molecules under steady-state conditions. As a result, it is found that vibrations play an important role in CISS, as has been suggested in several other theoretical reports on CISS, employing other models. It is interesting to see what this particular model has to say on the subject, and the analysis is well done and the results are thoroughly discussed. At the same time, it is not quite clear which new experimentally relevant insights this model can add. Based on the questions and comments below, it is not quite clear to me whether the publication criteria for JPCL are fulfilled.

We thank the reviewer for the positive comments and particularly for judging our results interesting. Following the referee's suggestions we improved the manuscript, adding a few lines of discussion and several new references, to better underline the relevance of our work to experimental data. However, CISS is to a large extent "terra incognita" and designing novel pathways of exploration, both experimentally and theoretically, is a helpful service to the community.

1) The model describes a linear chain, in which chirality is introduced by twisting p orbitals. Such electronic, as opposed to structural, chirality, is a fascinating topic, which has been studied, among others, by Solomon, Garner, and Corminboeuf. I would suggest acknowledging this in the manuscript.

This is a very useful comment as it made us to realize the need to better explain how chirality enters the model. Indeed, the adopted linear Hubbard chain is, by itself, non-chiral. In other terms, the adopted system is structurally non-chiral. However, electrochirality is introduced accounting for twisted on-site p-orbitals. This is an important concept that was somewhat overlooked in the original manuscript. This concept is now explicitly addressed on pages 4-5 "*As for the system, we consider the simplest model for correlated electrons, a linear Hubbard chain composed of N (typically 4) sites along the z axis. Structurally, the system is 4 non-chiral, but electronic chirality, or electrohelicity,39,40 is introduced accounting on each site for a single p-type orbital, perpendicular to z, but twisted along the chain so that the orbital on site i + 1 is rotated in the xy plane by an angle ϑi vs the orbital on site i, as shown in Fig.*

1a.," with proper reference to two papers from Solomon, Garner, and Corminboeuf (refs 39, 40) and is restated again on page 17

"*Results in Fig. 4 refer to a two-site Hubbard chain, a structurally non-chiral system, where chirality is enforced by the orbital twist."*

2) Experimental research on CISS so far has focused on structurally chiral systems. Why was a model system chosen here where chirality is only present on the electronic level, and what is the intended relation with the experiment?

Symmetry imposes that in real materials electronic chirality is associated with structural chirality, and therefore CISS experiment can only be performed on structurally chiral systems. However, our work suggests that it is the electronic chirality that governs the phenomenon, a quite intriguing issue that also shows up in the discussion of "chiral vibrations": the stretching mode of a two-site molecule is clearly non-chiral, but it acquires chirality due to the coupling to the chiral electronic system.

3) It is interesting to see what happens in connection with CISS when employing this particular model. It still remains unclear what this model can add to understanding the experiment that other previously studied models cannot.

This is the main criticism from the reviewer. As stated above, we believe that CISS is such a complex and so far poorly understood phenomenon, that any "interesting" hint towards its understanding is valuable. However, we took advantage from this criticisms trying to better highlight the connections between our theoretical results and the experiment. Specifically, we added a few sentences on page 13, and added several references to experimental papers 55-58.

4) In typical CISS experiments, the variation of polarization with bias voltage (which controls current) is relatively small (see,
e.g.,
https://eur01.safelinks.protection.outlook.com/?url=https%3A%2F%2Fdoi.org%2F10.1002%2Fsmll.202308233&data=05%7C02%7Canna.painelli%40unipr.it%7Cf4f479e3cb6f4687549c08ddc2ea1611%7Cbb064bc5b7a841ecbabed7beb3faeb1c%7C0%7C0%7C638881032848705057%7CUnknown%7CTWFpbGZsb3d8eyJFbXB0eU1hcGkiOnRydWUsIlYiOiIwLjAuMDAwMCIsIlAiOiJXaW4zMiIsIkFOIjoiTWFpbCIsIldUIjoyfQ%3D%3D%7C0%7C%7C%7C&sdata=s6lmW%2BAdlMfiNF0Re91Z3%2FG3WsR7sp20EynakxXLYJI%3D&reserved=0), at least compared to the strong increase with current seen here (e.g. Fig 1a). This should be discussed in comparison with the experiment.

This is an important point, that was unfortunately overlooked in the original submission. Indeed, an anomaly of the current anisotropy around V=0 is expected due to the vanishing denominator. Quite interestingly, several experimental results validate this anomaly and indeed it is explicitly discussed in Ref. 57. We address this topic in the manuscript (pg 13): "*We notice that the spin polarization has a smooth evolution with a current, while the current anisotropy shows anomalies around V = 0. This anomaly is intrinsic to a quantity whose denominator vanishes at V = 0. Strong non-linearities of G around V = 0 have indeed been experimentally observed.55–58*"

5) The finding that moving away from half filling and weak bonds promote polarization are interesting, but how do they compare with existing experimental data?

The filling of molecules in a molecular junction depends on the circuit details, so that it is possible that experimental results refer to non-half-filled systems. It would be very interesting to analyze this feature from the experimental perspective. We addressed this important point "*On the positive side, results in Fig. 2c could explain the large current anisotropies measured in transport measurements: the number of electrons inside the molecular junction depends on the circuit details,54 and it is likely that in the steady-state regime the junction bears a number of electrons different from the isolated molecule."*

More generally, we hope that our work will help experimentalists to plan novel measurements that will prove or possibly disprove our results.

Overall, it appears that a lot of interesting things can be learned from this work on the behavior of this model, but it is less clear what can be learned for the experiment and for understanding CISS.

The language appears a bit odd in places ("renormalize way") amd has occasional grammar issues - it may make sense to proofread the manuscript accordingly.

"renormalize away" is often used in physics, but most probably should be avoided in an ACS journal. Accordingly, we have rephrased the relevant sentences: "*A unitary transformation allows to embed the Holstein vibrations, modulating on-site energies, into the Hubbard Hamiltonian via a renormalization of model parameters …… The unitary transformation does not work for Peierls vibrations,"*

Additional Questions:

Urgency: Moderate

Significance: Moderate

Novelty: Moderate

Scholarly Presentation: High

Is the paper likely to interest a substantial number of physical chemists, not just specialists working in the authors' area of research?: Yes

Reviewer: 3

Recommendation: This paper may be publishable, but major revision is needed; I would like to be invited to review any future revision.

Comments:

Report on jz-2025-01813t

Just like the authors point out, the CISS effect is an intriguing phenomenon which is under much debate as to how to understand the origin of the effect. Whether it is poorly understood may be a question of whether there exists any quantitatively predictable theoretical model for the phenomenon or not. I would certainly agree that there is yet no quantitive theory that provides the answers for any system, molecular or solid state, that has been used in experiments. On the other hand, there is a fair bit of qualitative understanding that one should not simply dismiss. Nonetheless, the CISS appears to be difficult to formulate in terms of simple effective models.

This is the quest of the authors to the manuscript at hand and one can say that, despite some bit of success they fail in most ways others do in convincingly reproducing the CISS effect. At least when looking the numbers obtained through the calculations. I understand the vertical scales on the figures as 10^-6, 10^-4, and 10^-1 for the current anisotropy. By the way, current anisotropy is a good to use in the context of transport since, and I agree with the authors, the spin-polarization is not measured in this set-up. For the photoemission measurements, on the other hand, it is the spin-polarization that is measured which is why the terms makes sense in this set-up. Nonetheless, 10 % of current anisotropy is a good number.

Since the numbers do not match with experiments very well, except for some special conditions, which does not satisfy the authors – I praise this honesty, something which is not common these days – raises the question of whether there is physical insight enough to warrant publication of the results. On the other hand, we cannot blindly stare at the numbers. We should also be aware that negative results are quite important and for this reason, however, not only, I suggest that the paper should be published. A second reason is that the authors propose an alternative approach to the problem in which both direct Coulomb interactions and coupling to nuclear vibrations are included, yet allowing for simulations of non-equilibrium conditions. This is valuable and gives good cause for exploring the CISS effect using this approach.

Because, electron interactions would necessarily be part of the mechanism behind the CISS effect. There is nothing else in the set-up that can generate an anisotropy of the kind that is repeatedly measured by various groups using a wide range of conditions and a wide range of chiral molecules. Hence, although this paper is not the first attempt in which electron correlations are incorporated in the modeling, it is an important contribution and builds in the direction in which further research should be going.

Nonetheless, there are a few issues that the authors should address before the paper can be published.

We really enjoyed reading the statements by the reviewer. The reviewer has precisely understood the way we approach the problem, not pretending having solved the CISS conundrum, but being confident that we add some safe (either positive or negative) results that will help the community to build a more complete understanding of this intriguing phenomenon.

1) In the abstract, and also elsewhere in the paper, the authors mention that the CISS effect cannot be obtained in a two site electronic system. This would have to be a trivial statement unless I am missing something. At least four sites are necessary for the construction of a chiral chain of sites. What is the intention with stating the, what I perceive as a, trivial and obvious fact?

Also based on the comments from reviewer 2, we realized that in the original manuscript we failed to properly address the important topic of how chirality enters our model. Indeed, irrespective of the number of sites, the linear Hubbard chain we are considering is clearly non-chiral from a structural perspective. “Electronic chirality” (or electrohelicity as defined in ref. 39,40) enters the model with twisted on-site p-orbitals. In these conditions, also a two site chain becomes chiral. Indeed, several models have been proposed in the literature discussing two or three-site model (see e.g. refs 28, 29, 53, 62 and 64 in the manuscript), where chirality enters just thanks to an ad hoc choice of model parameters (typically SOC): in our model, chirality and the resulting SOC interactions are consistently derived from the orbital twisting. That said, we underline that in the two-site “electrochiral” system, spin polarization is forbidden due to the obvious lack of next-nearest neighbor interactions. Indeed, a well-known gauge transformation (that is rediscussed and extended in ESI to the current-carrying Hamiltonian and to Holstein coupling) demonstrates that spin polarization cannot be observed in in a linear Hubbard chain the lack of next-nearest neighbor

interactions. Therefore, the observation of CISS in a two-site (electrochiral) system, in the presence of out of equilibrium Peierls vibrations is a quite impressive result with important implications. We took advantage from the reviewer comments to improve the manuscript as to make our approach to chirality more transparent. Specifically, we modified the last sentence in the abstract (that was indeed confusing) to read "*Peierls vibrations play a special role: CISS cannot be observed in a Hubbard chain in the lack of next nearest neighbor interactions, but an out of equilibrium stretching mode in a two site chain leads to finite polarization"*. Moreover we rewrote several sentences in the introduction to clarify how chirality enters the model "*As for the system, we consider the simplest model for correlated electrons, a linear Hub bard chain composed of N (typically 4) sites along the z axis. Structurally, the system is 4 non-chiral, but electronic chirality, or electrohelicity,39,40 is introduced accounting on each site for a single p-type orbital, perpendicular to z, but twisted along the chain so that the orbital on site i + 1 is rotated in the xy plane by an angle ϑi vs the orbital on site i, as shown in Fig. 1a*.,"

2) In the literature, as well as in the present paper, the fact that the spin-orbit coupling is small is perceived as a problem concerning the spin-polarization and current anisotropy. However, there is no contradiction in this relation since spin-orbit coupling cannot on its own spin-polarize, it can only mix states. On the one hand, spin-orbit coupling is necessary since it couples the structure to the electronic spin. On the other hand, however, since the spin-orbit coupling typically is included at the single-electron level, and since it is not magnetic field-like, the result is a mixing of states, nothing else. In addition, interactions may enhance the effect of the spinorbit coupling. I think the author should discuss this misunderstanding in the field and also comment on that the spin anisotropy has to originate from interactions in one or another way.

We fully agree with the reviewer: SOC is necessary for CISS, but it is not enough. In the first place we need to break time-reversal symmetry (e.g. by introducing dissipation), or, as stated in the main text, we need out-of-equilibrium conditions, as obtained with moving electrons. As extensively discussed in the reference suggested by the reviewer (ref 16 of the revised manuscript) these two ingredients (SOC of the proper form+time-reversal symmetry breaking) allow for CISS responses, whose magnitude however is typically extremely small. And we again agree with the reviewer: to amplify CISS we need interactions. Electron correlations or electron vibration coupling both play a role in this respect, and most probably to really understand CISS both effects must be accounted for at the same time. A complex problem, that can only be tackled in simplified models: our contribution here is just a first step in this direction. To clarify this point we modified the intro "*CISS requires the breaking of time-reversal symmetry, as guaranteed by traveling elec*

trons, and well as the breaking of the spin-degeneracy, as made possible by spin orbit coupling (SOC).16 In organic molecules, SOC is tiny,9,17 and several strategies for its amplification, as needed to explain experimental data, have been proposed, relying on strong electron electron interactions,16,18,19 electron-vibration coupling,20,21 quantum interference,22 pola ronic effects,23–25 geometrical curvature in helical atomic chains,26,27 the close proximity to conical intersections,28–30 and dephasing phenomena,16,31 to cite just a few examples".

3) The authors neglect the spin-flip part of the spin-orbit coupling, something which is a trouble. By referring to a paper, which is also already old in this context, where this was a result of simulations, the authors take for granted that this assumption is safe. I disagree, both by that it is an incorrect approach to clarify the physics of a phenomenon, that with the author's words is poorly understood, and also since there is nothing ad hoc that suggests the correctness of such omission. Even so, DFT simulations cannot be used as telling the right answers to a question that, thus far, cannot be addressed using DFT. In fact, it was discussed in a recent paper [J Phys Chem Lett, 16, 4346 (2025)] that the transverse spin components, hence, spin-flip mechanisms are not negligible at all. I think that the authors should reconsider their simulations with the transverse spin-orbit coupling components included as well. Physically, one should expect the spins in the structure to be non-collinear and, therefore, reducing to a collinear approach is just not good enough. It can also be argued on good grounds that the CISS effect has to do with transfer of angular momentum within the structure, something that seems to go missing with the current assumptions.

In the adopted model, where (electro)chirality is due to tilted on-site pi-orbitals in a linear chain, the spin-flip terms of SOC exactly vanish, simply because the electron orbital momentum is parallel to the z-direction (the axis chain). So, introducing spin-flip SOC terms within this model would be inconsistent. However, we recognize that spin-flip terms may not be as innocent as often maintained in the literature (many papers have been published neglecting spin-flip SOC interactions, see e.g. refs 19, 21 53 and 62 in our manuscript). Indeed, we are currently working on a slightly more elaborate model, where, arranging the atoms in a truly 3D chiral structure, we can account for all SOC terms, including those responsible for spin-flips. This issue is shortly addressed in the manuscript ""*In the adopted model, the electron orbital momentum is aligned along the chain and the SOC interaction necessarily preserves the z component of the total spin, ˆ Sz, so that spin-flips are not accounted for. This is an inherent feature of the proposed model, but investigating the role of spin flips in CISS would definitely be interesting, particularly in view of the contrasting opinions in recent literature.16,42"*

Just below the assumption of collinear spin-orbit coupling, there is a mention of the S_z subspace. It is not clear what S_z refers to, although I interpret it as being related to the total spin of the molecule.

Thanks, we have now properly defined S_z as the z component of the total spin.

4) The authors claim that spin-orbit coupling between next nearest sites is sufficient to generate spin-polarization. However, spinorbit coupling can by itself not generate spin-polarization, it has to be presence of the electron interactions that makes the difference. Overall, I urge the authors to emphasize the crucial role interactions have for a meaningful modeling of the system to address the CISS effect.

Next nearest neighbor interactions (either hopping or SOC) are a prerequisite for CISS in simple linear Hubbard modes. This results from a well-known gauge transformation that we re-derive in ESI, with the inclusion of current and Holstein vibrational coupling. As already discussed in the answer to comment 2, the presence of SOC as well as of next-nearest neighbor (SOC or hopping) interactions are a necessary condition to observe CISS in **traveling** electrons, i.e. in out-of-equilibrium conditions. We have now clearly stressed these points in the revised manuscript. This however does not mean that sizable CISS responses are seen, far from it! Indeed, other interactions, including electron correlations and/or electron-vibration coupling are for sure needed to observe sizable CISS responses. And it is precisely this that makes the problem complex and asks for the definition of simplified models, the avenue along we are trying to move with tis manuscript.

In summary, the paper should be published provided that the authors address the questions raised above in a serious way.

Additional Questions:

Urgency: High

Significance: High

Novelty: High

Scholarly Presentation: High

Is the paper likely to interest a substantial number of physical chemists, not just specialists working in the authors' area of research?: Yes
